# Detection of *Mycobacterium avium* ssp. *paratuberculosis* in Cultures From Fecal and Tissue Samples Using VOC Analysis and Machine Learning Tools

**DOI:** 10.3389/fvets.2021.620327

**Published:** 2021-02-03

**Authors:** Philipp Vitense, Elisa Kasbohm, Anne Klassen, Peter Gierschner, Phillip Trefz, Michael Weber, Wolfram Miekisch, Jochen K. Schubert, Petra Möbius, Petra Reinhold, Volkmar Liebscher, Heike Köhler

**Affiliations:** ^1^Institute of Mathematics and Computer Science, University of Greifswald, Greifswald, Germany; ^2^Institute of Molecular Pathogenesis, Friedrich-Loeffler-Institut, Jena, Germany; ^3^Department of Anaesthesia and Intensive Care, University Medicine Rostock, Rostock, Germany; ^4^National Reference Laboratory for Paratuberculosis, Institute of Molecular Pathogenesis, Friedrich-Loeffler-Institut, Jena, Germany

**Keywords:** bacterial culture, diagnostics, machine learning, *Mycobacterium avium* ssp. *paratuberculosis*, paratuberculosis, random forests, variable selection, volatile organic compound

## Abstract

Analysis of volatile organic compounds (VOCs) is a novel approach to accelerate bacterial culture diagnostics of *Mycobacterium avium* subsp. *paratuberculosis* (MAP). In the present study, cultures of fecal and tissue samples from MAP-infected and non-suspect dairy cattle and goats were explored to elucidate the effects of sample matrix and of animal species on VOC emissions during bacterial cultivation and to identify early markers for bacterial growth. The samples were processed following standard laboratory procedures, culture tubes were incubated for different time periods. Headspace volume of the tubes was sampled by needle trap-micro-extraction, and analyzed by gas chromatography-mass spectrometry. Analysis of MAP-specific VOC emissions considered potential characteristic VOC patterns. To address variation of the patterns, a flexible and robust machine learning workflow was set up, based on random forest classifiers, and comprising three steps: variable selection, parameter optimization, and classification. Only a few substances originated either from a certain matrix or could be assigned to one animal species. These additional emissions were not considered informative by the variable selection procedure. Classification accuracy of MAP-positive and negative cultures of bovine feces was 0.98 and of caprine feces 0.88, respectively. Six compounds indicating MAP presence were selected in all four settings (cattle vs. goat, feces vs. tissue): 2-Methyl-1-propanol, 2-methyl-1-butanol, 3-methyl-1-butanol, heptanal, isoprene, and 2-heptanone. Classification accuracies for MAP growth-scores ranged from 0.82 for goat tissue to 0.89 for cattle feces. Misclassification occurred predominantly between related scores. Seventeen compounds indicating MAP growth were selected in all four settings, including the 6 compounds indicating MAP presence. The concentration levels of 2,3,5-trimethylfuran, 2-pentylfuran, 1-propanol, and 1-hexanol were indicative for MAP cultures before visible growth was apparent. Thus, very accurate classification of the VOC samples was achieved and the potential of VOC analysis to detect bacterial growth before colonies become visible was confirmed. These results indicate that diagnosis of paratuberculosis can be optimized by monitoring VOC emissions of bacterial cultures. Further validation studies are needed to increase the robustness of indicative VOC patterns for early MAP growth as a pre-requisite for the development of VOC-based diagnostic analysis systems.

## Introduction

Detection of volatile organic compounds (VOCs) derived from bacterial metabolism has been proposed as a novel approach in diagnostic microbiology. VOCs originate from metabolic processes of the bacteria. Due to their physicochemical properties, they transform into gaseous state already at low temperatures. Appearing in very low concentrations (nmol/L—pmol/L or ppbV—pptV) they belong to all classes of organic substances (1). Technologies in use for the analysis of volatiles include (high-resolution) mass spectrometry (MS) approaches, including soft chemical ionization mass spectrometry (SCIMS) or gas chromatography-mass spectrometry (GC-MS), spectroscopic techniques, and sensors. Analyzers can be allocated to two categories, namely *offline* systems, which require sample workup such as pre-concentration prior to analysis, and *online* instrumentation, which can analyze samples directly without manipulation (2). Online monitoring of bacteria-specific VOC-profiles during cultivation would enable direct species identification without further processing of samples, and would thus reduce labor and costs. In addition, highly sensitive detection of VOCs released by growing bacteria could allow detection of bacterial growth earlier than currently possible. This is of special interest for slow-growing bacteria, such as *Mycobacterium avium* ssp. *paratuberculosis* (MAP).

Bacterial culture on solid or liquid media with subsequent species confirmation via polymerase chain reaction (PCR) is still considered the most sensitive and robust diagnostic method for the detection of MAP in different types of samples (3). This labor-intensive and time-consuming procedure takes weeks to months until reliable results are available (4). Automated liquid culture systems, which were adopted recently for MAP, resulted in reduced cultivation times, but still demand further processing of the samples for species identification (5, 6). In an attempt to reduce time to result, (real-time) PCR based techniques have been established and introduced in routine diagnostics (7–9). The performance of PCR based methods depends largely on the efficacy of the protocol used for nucleic acid extraction from clinical samples (10, 11). The detection rate is reduced when samples with low bacterial load are tested (12, 13). On the other hand, due to their high analytic sensitivity, these methods are prone to sample misclassification by false positive results because of cross contaminations (own unpublished results). The main advantage of PCR techniques compared to bacterial culture is the short time necessary until results are available. A diagnostic approach combining the advantages of both techniques without increased risk of misclassification is highly desirable.

Recent studies have shown that it is possible to detect growth of MAP by measuring volatile organic compounds in the headspace of bacterial cultures (14, 15), even before colonies become visually apparent (16). Instead of individual indicative substances, these studies recorded a selection of several VOCs (i.e., a “VOC profile”) in order to differentiate growing MAP cultures from control vials and from cultures of other mycobacterial species. The composition of the VOC profiles varied to some extent depending on MAP strain (14, 15), culture medium (15), bacterial density (14, 15), and duration of incubation (15). However, it was possible to define a core profile of 28 VOCs related to growth of MAP cultures by a meta-analysis (17).

As a common feature of these studies, pure bacterial cultures were grown using laboratory strains of different field isolates. In practical diagnostics, however, MAP is being isolated from different matrices, such as feces and tissue samples of variable animal species, solid or liquid manure and even dust from the housing environment of the animals. These matrices may emit additional VOCs during cultivation, which might possibly interfere with the MAP-specific VOC profile. This problem has not been addressed so far (17).

Matrix-related VOC emissions were investigated in this study as a necessary step toward practical application. Cultures of native diagnostic samples from MAP infected and non-suspect cattle and goats were examined to elucidate the effects of the sample matrix (feces or tissue) and of the animal species on VOC emissions during cultivation. On this basis, the applicability of the MAP-specific core-profile to diagnose MAP cultures was reviewed.

Previous studies showed that VOC concentrations above MAP cultures varied in relation to bacterial density (14, 15). The majority of substances increased with increasing bacterial counts, others decreased, or they decreased after an initial increase (15). Therefore, a data analysis workflow based on random forests was developed to capture those varying VOC patterns in a multivariate fashion. The workflow comprises also a random forest-based variable selection procedure to pick all relevant VOCs from the full panel of volatile compounds that were detected in the headspace volume of the bacterial cultures. Repeated cross-validation was deployed to robustify the results of the workflow.

We analyzed the data, on the one hand, focusing on MAP presence and, on the other hand, focusing on different stages of MAP growth in native samples, taking into account varying patterns of VOC emission in relation to bacterial growth. Thus, by using a tailored machine learning workflow, we aimed at identifying MAP-specific VOC profiles that allow sample classification already after short periods of cultural incubation.

## Materials and Methods

### Samples

Fecal and tissue samples (*n* = 80) with culturally pre-defined MAP status were derived from the sample collection of the German National Reference Laboratory for paratuberculosis at the Friedrich-Loeffler-Institut. Fecal samples from cattle and goats originated from different animals and herds enrolled in a field study performed in 2016 and 2017. The study protocol was approved by the responsible authority, the Animal Health and Welfare Unit of the “Thüringer Landesamt für Verbraucherschutz” (permit number 04-102/16, date of permission: 20.04.2016). Goat tissue samples (mesenteric lymph nodes, tissue from ileum or jejunum) were obtained from different goats necropsied in the course of an experimental infection trial in 2011 and 2012. The animal experiment was approved by the responsible authority (see above, permit number 04-001/11, date of permission: 03.03.2011). Cattle tissue samples were collected after slaughter from different cattle during a slaughterhouse survey in 2007 (18). Presence or absence of MAP was originally examined after admission to the laboratory by cultural isolation following standard laboratory procedures. After first processing, the samples were stored at −20°C (cattle and goat feces, goat tissue) and −80°C (cattle tissue) until preparation for the present study. An overview of the samples is given in Table 1. The MAP isolates obtained from cattle and goat feces and from cattle tissue represent eight different MAP genotypes (see Supplementary Table 1). The MAP isolates from goat tissue were all derived from MAP strain JII-1961 (19), which was used for inoculation of the animals in the experimental infection trial.

**Table 1:** Overview of the samples included in the study.

		**MAP-negative**	**MAP-positive**
**Matrix**	**Species**	**Number of Herds/ Animals/Samples**	**Number of Herds/ Animals/Samples/ MAP genotypes**
Feces	Cattle	5/10/10	4/10/10/3
	Goat	3/10/10	3/7/10/2
Tissue	Cattle	1/2/10	5/5/10/6
	Goat	1/5/10	1/5/10/1

*Numbers represent the number of herds, number of animals, number of samples and number of MAP genotypes, respectively, for each set of samples (for details on the genotypes see Supplementary Table 1)*.

### Sample Preparation

The procedures followed in this study conform to protocols established in previous studies (15, 17) in order to enable comparability.

To prepare the test tubes for a fecal sample, 3 g of feces were decontaminated in 30 mL of 0.75% hexadecylpyridinium chloride (HPC, Merck, Darmstadt, Germany) for 48 h in order to eliminate non-MAP flora (20). The supernatant was discarded and the sediment (1–2 mL) was further processed as described below. The tissue samples originated from different parts of ileum, jejunum, or mesenteric lymph nodes. After separating tissue and fat, approximately 1 g of tissue from different parts of the sample were gathered. Decontamination was performed with 0.9% HPC for 24 h at room temperature. The tissue samples were centrifuged and the sediment resuspended with 1 mL of sterile phosphate buffered saline (PBS) to maintain a physiological pH (20).

For both fecal samples as well as tissue samples, nine tubes of slanted Herrold's Egg Yolk Medium with Mycobactin J and Amphotericin, Nalidixic Acid and Vancomycin (HEYM, Becton Dickinson, Heidelberg, Germany) were inoculated with 200 μL of the resulting sediments. After spreading the inoculum evenly over the surface of the solid medium, the tubes were incubated at about 37°C under aerobic conditions. For each set of samples (goat or cattle, negative or MAP-positive), inoculation was performed at a separate day to eliminate carry-over effects. Parallel to these samples, 30 control tubes were prepared for each set either with 200 μL of 0.75% HPC (for feces) or with 200 μL of a 1:1 mixture of PBS and 0.9% HPC (for tissue) without fecal or tissue matter. The control tubes were treated and incubated under the same conditions as the test tubes.

Colony growth was assessed regularly by visual inspection, colony counts up to 50 colonies were counted, higher colony counts were estimated following a standard laboratory procedure. Growth was scored at the end of the pre-determined incubation period in the following way: score 0—no growth visible, score 0.5—one to 20 colonies, score 1−21 to 50 colonies, score 2−51 to 100 colonies, score 3—loose layer, score 4—dense layer. The duration of culture incubation was defined depending on the expected growth characteristics of the MAP isolates in order to cover different growth stages of the individual samples. Of the nine test tubes per original sample, three were randomly selected at the pre-determined end of the incubation period after 4, 6, and 8 weeks for cattle feces and tissue and goat tissue, and after 16, 18, or 20 weeks for goat feces. An exception had to be made for MAP cultures from goat feces: The cultures of two samples grew unexpectedly fast. Incubation of three randomly selected culture tubes was therefore interrupted after 4, 6, and 8 weeks and the tubes were moved to a refrigerator to limit further growth. Before GC-MS measurement, these tubes were again incubated for 7 days at 37°C. Finally, they were measured 16–20 weeks after inoculation. The test tubes of the other MAP-positive and negative samples and the control tubes were incubated for the pre-determined period of 16, 18, or 20 weeks. The final sample sizes can be seen in Table 2.

**Table 2:** Sample sizes for VOC analysis per species and matrix with regard to incubation periods in accordance with the study design (4/6/8 weeks in general and 16/18/20 weeks for goat feces, respectively).

**Matrix**	**Species**	**Control vials**	**MAP-negative**	**MAP-positive**
Feces	Cattle	20/20/20	20/20/20	18/20/20
	Goat	20/18/20	20/20/20	20/20/20
Tissue	Cattle	20/20/20	20/20/20	20/20/20
	Goat	20/20/20	20/20/20	20/20/20

### VOC Analysis

The headspace volume of the tubes was sampled by means of needle trap microextraction (NTME) and analyzed by GC-MS as described elsewhere (14, 15). The GC-MS system consisted of an Agilent 7890A gas chromatograph and an Agilent 5975C inert XL MSD mass spectrometer. In order to identify unknown VOCs from the mass spectra, first, a mass spectral library search (NIST 2005 Gatesburg, PA, USA) was carried out and, subsequently, compounds were verified and quantified by measurements of pure reference substances. Altogether, more than 100 volatile substances were detected in the headspace volumes. VOCs which could not be identified unequivocally, which could not be quantified or which were assigned to contamination from room air were excluded from the VOC panel in a pre-processing screening of the GC-MS spectra.

### Data Analysis

Exploratory data analysis included heat maps to visualize normalized concentrations of each VOC in the individual samples, and principal component analysis (PCA) to assess if differentiation of MAP-positive and negative samples is possible in general. Basic graphical representations of the data (e.g., box-whisker plots, scatterplots) were explored interactively by means of a specially tailored R Shiny app. A correlation analysis using Spearman's rank correlation coefficient was performed for VOC measurements of bacterial cultures with visible growth to detect clusters of compounds with similar or opposite trends which might be related to MAP growth.

VOC emissions of control vials were considered baselines and used for quality assessment. Effects of the extended incubation period of 16–20 weeks in comparison to 2–8 weeks on VOC concentration in the headspace volume above pure media was assessed using two-sided Mann–Whitney-*U*-tests with Bonferroni *p*-value correction. A tentative screening for potential influences from exogenous sources was performed by assessing variations of control vials between different days of inoculation (using Kruskal–Wallis tests with Bonferroni *p*-value correction) and comparing concentration levels of control vials with those of actual samples (using two-sided Mann–Whitney-*U*-tests with Bonferroni correction; details in Supplementary Table 4).

In order to assess which VOCs might originate from traces of original sample material, feces or tissue, VOC concentration of MAP-negative test tubes was compared to control vials prepared at the same day using one-sided Mann–Whitney-*U*-tests with Benjamini–Hochberg *p*-value correction. We deployed a one-sided test to capture only VOCs with higher concentration values above MAP-negative test tubes compared to control vials.

Identification of MAP-specific VOC emissions was tackled using machine learning tools: Since the absence or presence of MAP was known for each sample and MAP growth had been scored for each VOC measurement, both could be used as targets for a supervised learning task. The objective of our workflow was to classify samples based on their VOC measurements with high accuracy and to identify VOCs supporting the classification. We decided to base our approach on random forests to be able to consider arbitrary patterns of multiple VOCs in combination. Random forests are completely data-driven and do not assume a specific underlying distribution of the data. In brief, a random forest classifier consists of a large number (typically several hundreds) of decision trees (21–23). Hence, their results are always aggregated across their decision trees, as an inspection of individual trees is not insightful. One result that can be drawn from random forests is a ranking of variable importance. The importance of a variable is determined for each decision tree using the observations that had not been used to construct the respective tree and scored by the loss of classification accuracy after resampling the measurements of the variable. This approach is based on the idea that an informative variable contributes considerably to the classification accuracy of a decision tree and thus resampling of an informative variable will lead to a high loss in accuracy, whereas resampling of a non-informative variable will hardly affect the classification accuracy. The loss of accuracy for each variable is reported as average across all decision trees of the random forest.

The variable selection algorithm Boruta (24) was used to reduce the set of VOCs to those that show variations related to MAP presence or growth. The Boruta algorithm uses random forest variable importance measures to compare variables with randomly permuted copies of themselves. Only if an original variable outperforms the best among all copies it is considered important and used further.

**Figure 1:**
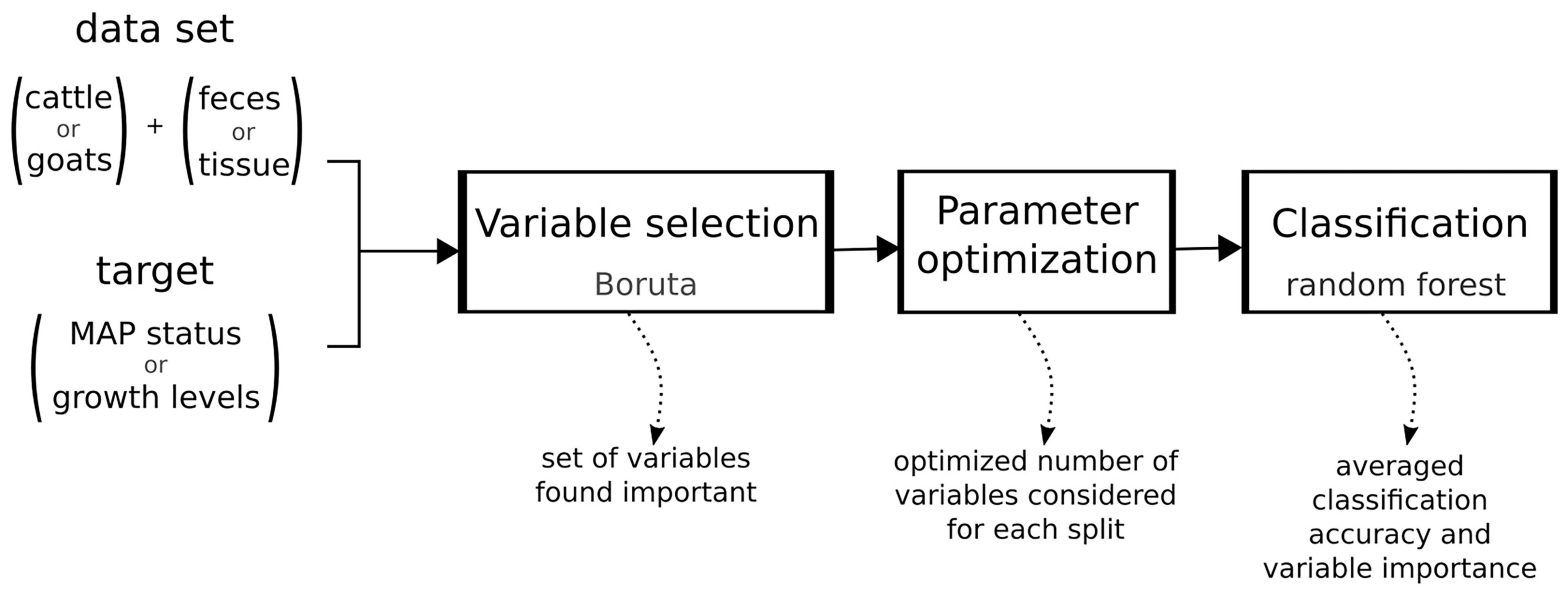
Machine learning workflow.

Using the methods described above a robust machine learning workflow was set up as follows (Figure 1):

Step 1: *Variable selection with Boruta*. To decrease variance of the decision, the algorithm was applied 30 times and only variables found important in more than 27 of the iterations were used further.

Step 2: *Parameter optimization for random forest*. We optimized the number of variables considered for a split in a decision tree (parameter mtry) to maximize classification accuracy.

Step 3: *Classification using random forest*. A random forest classifier consisting of 500 decision trees using the variables selected in step 1 and the optimized parameter from step 2 was trained and the results averaged over 10 repeats of 10-fold cross-validation. For each VOC used in classification the importance measure is the mean decrease in accuracy when randomizing the values of that VOC.

The caret package (25) was used to streamline steps 2 and 3 such that the parameter optimization used the same cross-validation sets as the final classification. As the Boruta algorithm is not yet implemented in the caret package, the variable selection process is based on the complete data set outside of cross-validation.

This workflow was applied to address the two central objectives of the study, first, classification of MAP-positive vs. negative samples to find VOCs specific to MAP presence, and second, differentiation between the different stages of growth (scores from 0 to 4 as described above) and negative samples to find VOCs indicative of the stages of bacterial growth and thus possible candidates enabling accelerated cultural detection.

These analyses were performed for both species and both sample matrices separately. For the growth classifiers, the data was distributed unevenly over different growth stages and upsampling was applied for balancing, except for growth scores that were not observed for a set of samples. To summarize the results, we report the number of selected variables (step 1), the optimized number of variables considered for each new split (step 2) and the averaged classification accuracy of the final model (step 3).

The workflow was implemented in R *v3.6.2*. (26) with packages Boruta *v6.0.0* (24) and caret *v6.0-86* (25), which depends on the package randomForest (27). Packages used for data manipulation were dplyr (28) and tidyr (29), and packages used for data visualization were ggplot2 (30), pheatmap (31), factoextra (32), corrplot (33), ggridges (34), ggstance (35), plotly (36), and shiny (37).

## Results

### VOC Panel

**Figure 2:**
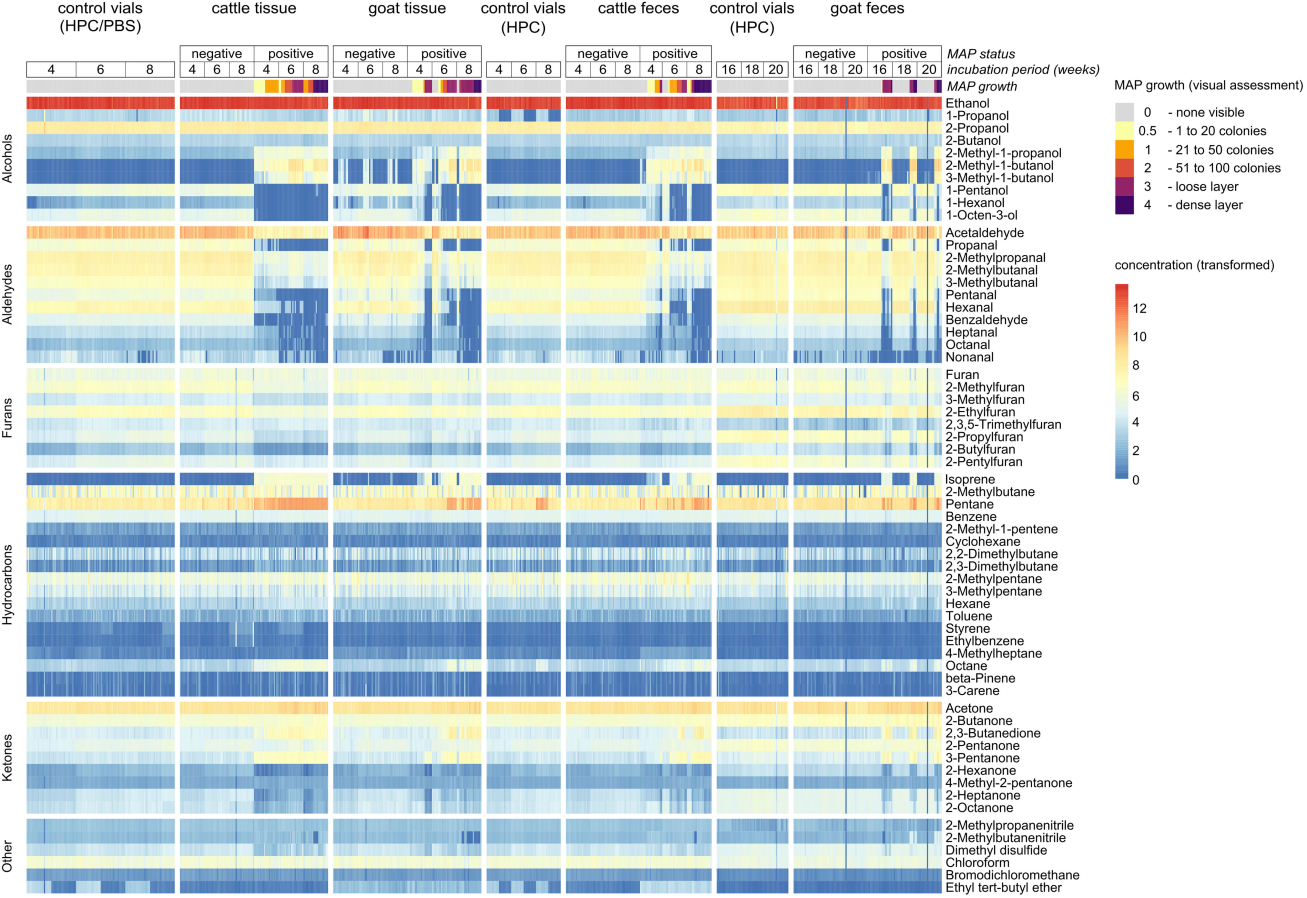
Heat map including all 62 VOCs and all samples. Concentration values are normalized via log(1+x)-transformation for better visualization.

VOC analyses resulted in a panel of 62 volatile substances (Supplementary Tables 2, 3). They belong to the classes of hydrocarbons including acyclic hydrocarbons, alcohols, ketones, aldehydes, furans, nitriles, organosulfur compounds, halogenated hydrocarbons, and ethers. Visual data exploration revealed that some of these compounds showed distinctive differences in concentration for MAP-positive samples in comparison to negative samples and control vials. This became evident in the heat map including all VOCs and all samples (Figure 2) and also in the visualization based on principal component analysis (PCA, Supplementary Figure 1). Not only increased, but also decreased concentrations above MAP-positive cultures were observed (Figure 2). Some of the MAP-positive goat feces samples did not show any bacterial growth, even after 20 weeks of incubation, which is very likely the reason why their VOC composition resembles negative samples in these visualizations. Correlation analysis revealed clusters of highly correlated compounds in the headspace of bacterial cultures with visible growth (Supplementary Figure 2).

The extended incubation period of 16–20 weeks affected most of the compounds (both increase and decrease in concentration, Supplementary Table 4). Tentative screening for VOCs from exogenous sources captured a single compound: Ethyl tert-butyl ether (ETBE) showed increased levels on 3 consecutive days for both control vials and test tubes irrespective of the content of the test tube (Supplementary Figure 3 and Supplementary Table 4). This compound is a fuel additive and therefore most likely contamination from laboratory room air. Thus, ETBE was excluded from the VOC panel as it introduced a systematic bias.

### VOCs Originating From Feces or Tissue

VOC emissions from sample material were analyzed by comparing measurements of negative samples with control vials (see Supplementary Table 5). Fecal samples showed significantly higher concentrations of cyclohexane than control vials, whereas tissue samples showed significantly higher concentrations of acetaldehyde and 1-propanol. Cattle samples were characterized by higher levels of ethanol, propanal, 2-methylpropanal and acetone. In addition, cattle feces samples showed increased concentration levels of 2-propanol, and cattle tissue samples exhibited higher levels of furan, chloroform and 2-methylpropanenitrile. The latter was also elevated in goat feces samples, whereas goat tissue samples were characterized by 4-methylheptane, 2,3-butanedione, 2-methyl-1-butanol and 3-methyl-1-butanol. The last two compounds were detected above LOQ only in goat tissue samples, apart from MAP-positive samples.

### VOCs Indicating MAP Presence

**Figure 3:**
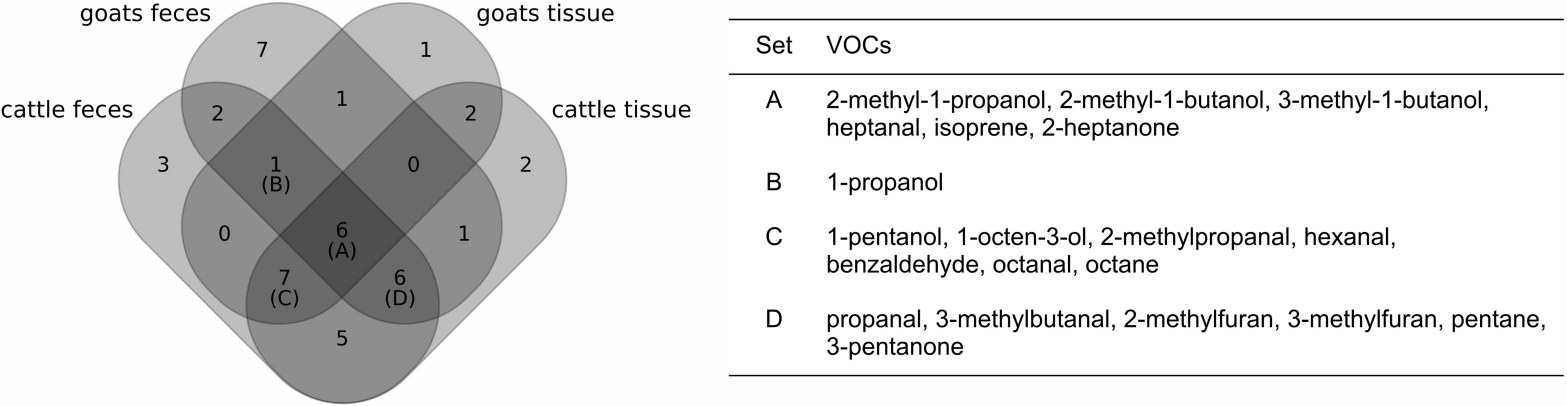
Comparison of the selection of VOCs indicating MAP presence for each set of samples (**left**: Venn diagram, **right**: table with details on the intersections of at least three sets). Set A represents the set of VOCs that were selected in all four settings, whereas sets B, C, and D represent sets of VOCs that were selected in three of four settings.

By comparing headspace VOC compositions of MAP-positive and negative samples by our random forest-based workflow, 44 of 61 VOCs were found to show indicative variations between these two groups in at least one of the four settings. The number of selected VOCs ranges from 18 VOCs for goat tissue to 30 VOCs for cattle feces (see Supplementary Table 6). Six compounds were selected in all four settings (Figure 3): 2-Methyl-1-propanol, 2-methyl-1-butanol, 3-methyl-1-butanol, heptanal, isoprene, and 2-heptanone. Further, 14 compounds were selected in three settings, comprising six aldehydes (propanal, 2-methylpropanal, 3-methylbutanal, hexanal, benzaldehyde, octanal), three alcohols (1-propanol, 1-pentanol, 1-octen-3-ol), two hydrocarbons (pentane, octane), two furans (2-methylfuran, 3-methylfuran), and one ketone (3-pentanone).

We observed that 3-methyl-1-butanol exhibited the maximum variable importance in two of four settings (for fecal samples). However, the relative variable importance values of the compounds varied considerably between the four settings (Supplementary Figure 4). While, three settings yielded a rather steep decline in variable importance from the top compound to the least informative compound of the selection, the results for cattle tissue samples showed that a large proportion of compounds reached comparatively high variable importance values. Ranking compounds by their respective importance value reflects the high variance in variable importance between different settings: Only 2-methylbutanal, pentanal and heptanal were consistently ranked among the top ten variables and selected in at least two settings. However, for all four settings, the random forest classifiers reached high accuracies in discriminating between negative and MAP-positive samples (cross-validated accuracy between 0.89 for goat feces and 1.00 for cattle tissue, Supplementary Table 6).

### VOCs Related to MAP Growth

**Figure 4:**
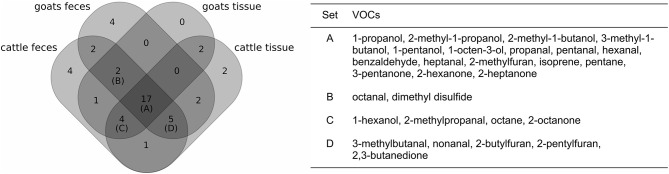
Comparison of the selection of VOCs related to MAP growth for each set of samples (**left**: Venn diagram, **right**: table with details on the intersections of at least three sets; see also Figure 3).

The refined analysis targeting the varying bacterial growth densities resulted in a similar selection of VOCs as before: 42 of the 44 VOCs that had been considered before were also selected to differentiate between levels of MAP growth in at least one of the four settings (Figure 4). Four VOCs were found to be related to MAP growth additionally. One of them, 2-pentylfuran, was selected in three of the four settings, whereas the other three compounds (2-pentanone, bromodichloromethane, 2-methylbutanenitrile) had been selected only once.

For each of the four settings, the number of selected compounds increased by four to eight compounds in the refined analysis (Supplementary Table 6). Thus, some VOCs were in total selected more often than in the previous analysis. 2-Hexanone and pentanal, which had previously passed the selection criteria only in one and two settings, respectively, were now included in all four settings. Acetaldehyde and octanal were excluded for the refined analysis regarding cattle tissue samples, while they had been selected for this setting in the previous analysis. However, both were selected in another setting (cattle feces and goat feces, respectively), for which they had not been considered in the previous analysis. Apart from these four compounds, the remaining 40 compounds of the previous analysis differed only in regard to a single setting.

An overview of the relative importance of selected VOCs per setting is given in Supplementary Figure 4. Due to different selections of VOCs and the redefined target of classification, relative importance values for the refined analysis deviate from those of the previous analysis. It should be noted that an increase in importance does not necessarily correspond to an increase in concentration and vice versa, as pictured in Supplementary Figure 5. Instead, the importance of a single compound for a specific level of bacterial growth should be considered in relation to the importance of other compounds in the same setting.

Cross-validated classification accuracies ranged from 0.82 for goat tissue samples to 0.89 for cattle feces samples (Supplementary Table 6). Misclassifications mainly occurred between related classes (e.g., between “MAP-negative” and “score 0,” but not between “MAP-negative” and “score 4,” Supplementary Tables 7–10).

**Figure 5:**
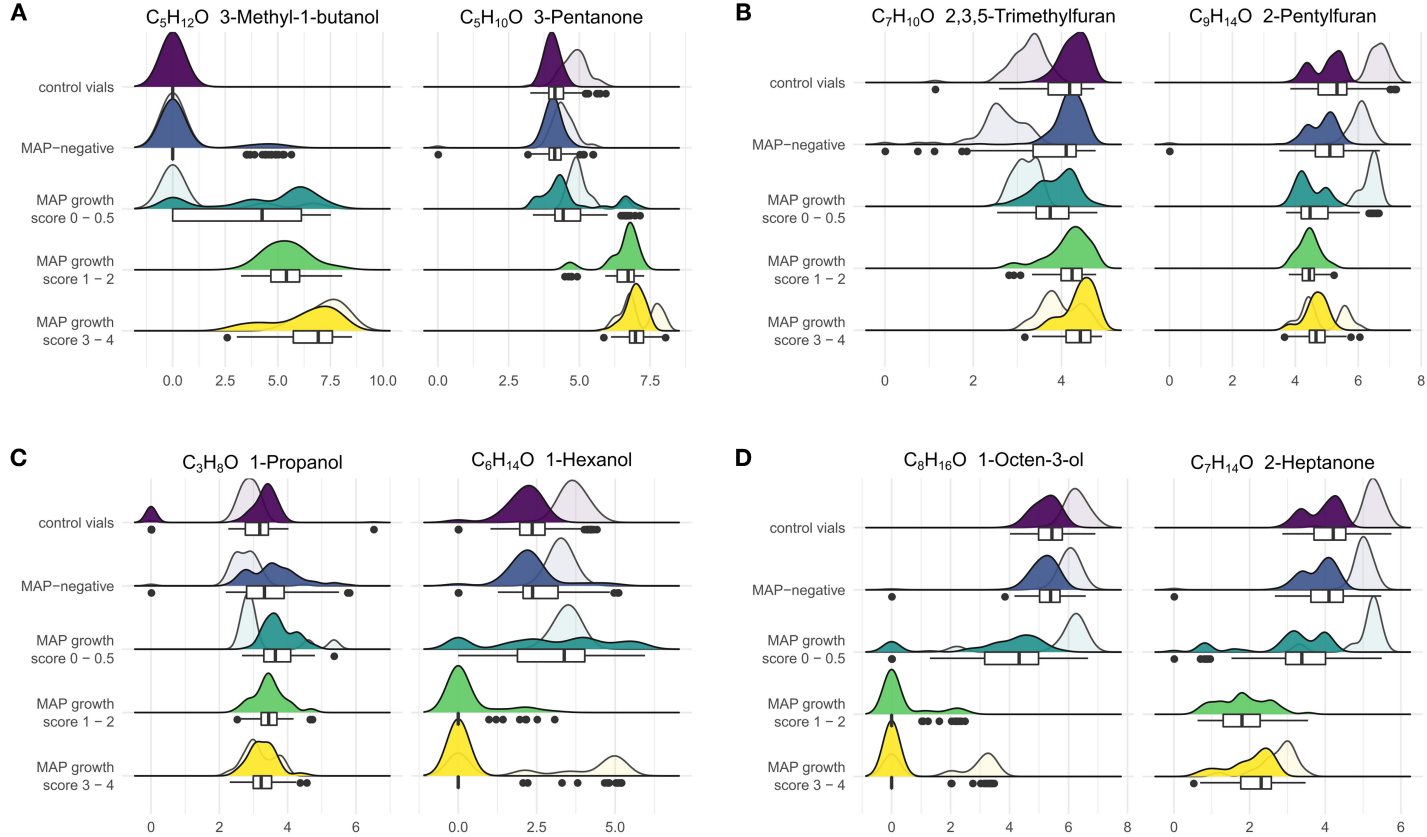
VOCs showed varying trends with increasing bacterial density. The figure combines box-whisker plots and smoothed histograms (filled: incubation period of 4, 6, and 8 weeks, transparent: incubation period of 16, 18, and 20 weeks). The x-axis indicates log(1+x)-transformed concentration values, and the y-axis indicates observed frequencies of the respective concentration values grouped by MAP status and growth scores (see section Sample Preparation). **(A)** VOCs with an increase in concentration in relation to MAP growth, **(B)** VOCs with a decrease for early bacterial growth and increase for higher bacterial densities, **(C)** VOCs with an increase for early bacterial growth and decrease for higher bacterial densities, **(D)** VOCs with a decrease in concentration in relation to MAP growth. Goat feces samples that did not show any bacterial growth after 20 weeks of incubation were excluded for this visualization. For further VOCs see Supplementary Figures 6–8.

Regarding VOCs which were included in at least two settings for classifying growth scores, compounds of some substance classes showed variable tendencies: Alcohols and ketones with up to five carbon molecules (except for 1-propanol and 1-pentanol) increased above growing MAP cultures, while substances of the same classes with higher carbon numbers (up to C8) decreased in concentration (Figure 5). The concentrations of hydrocarbons, including isoprene, but except for styrene, increased in the headspace of MAP cultures in comparison to control vials. All aldehydes showed a decrease in concentration with growing MAP cultures.

Furan concentrations did not differ markedly between negative and MAP-positive cultures and tended to be lower in the headspace of positive cultures. As an exception, concentrations of 2,3,5-trimethylfuran and 2-pentylfuran decreased above MAP cultures without visible growth or with few colonies (score 0–0.5) and slightly increased again for higher bacterial densities (score 1–4). Conversely, 1-propanol and 1-hexanol tended to increase above MAP cultures with score 0–0.5 and decreased with score 1–4. While, these changes were less pronounced for 1-propanol, 1-hexanol showed a steep decline below the limit of quantification from early phases of bacterial growth to higher bacterial densities (Figure 5).

**Figure 6:**
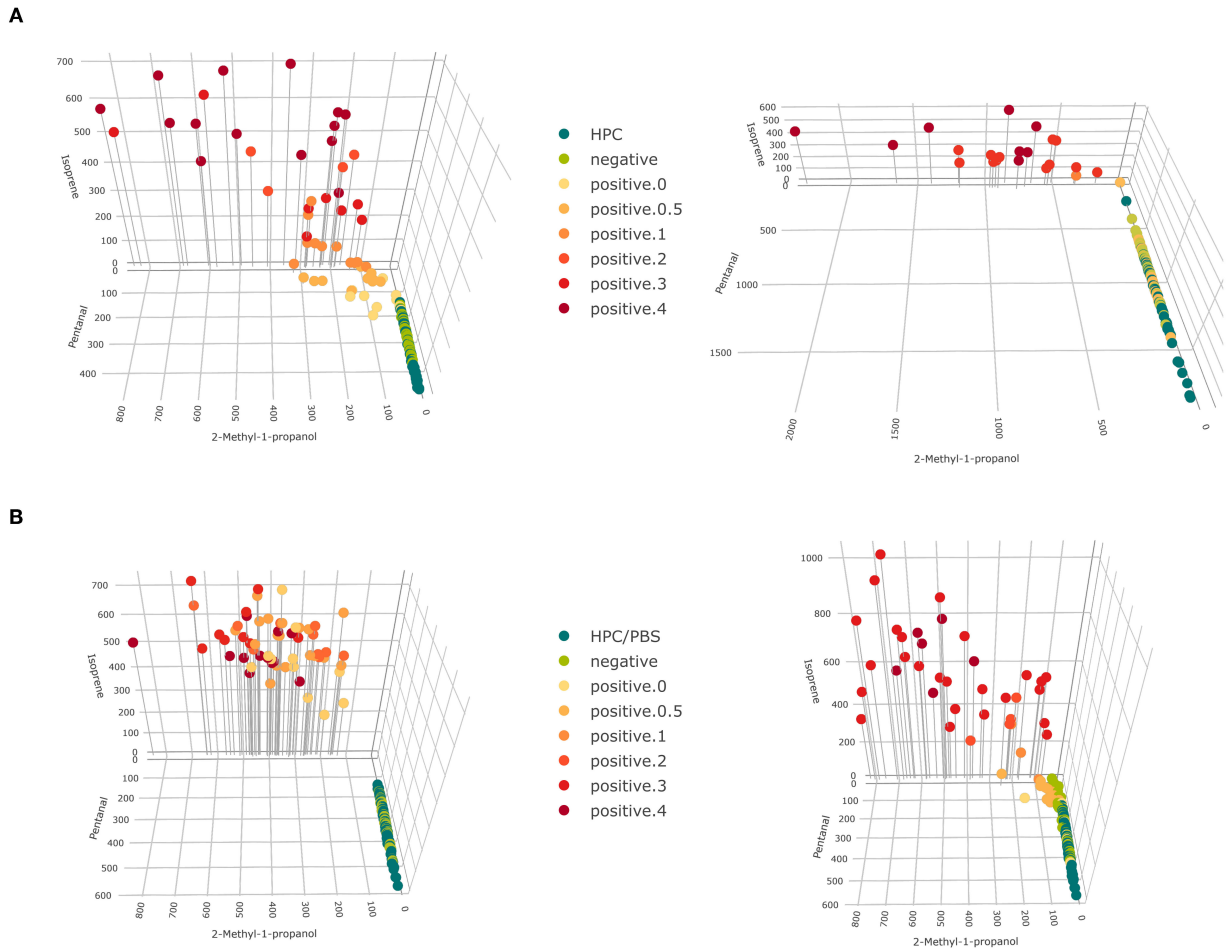
3D scatter plots for concentrations of 2-methyl-1-propanol, pentanal, and isoprene for **(A)** fecal samples and **(B)** tissue samples (**left**: cattle, **right**: goat). Colors represent levels of bacterial growth and drop down lines aid spatial visualization.

VOCs that have been selected in at least two settings for classifying growth scores have also been selected in at least two settings for classifying MAP presence, due to the higher selection frequency in the refined analysis. Thus, we consider the selection of VOCs presented in Supplementary Figures 6–8 as a final set of VOCs indicating growth of MAP cultures in the present study. This selection represents all relevant compounds for the present study, not a minimum selection. Indeed, stages of bacterial growth could be discriminated with few compounds, as pictured in Figure 6.

## Discussion

VOC measurements of biological samples are typically characterized by a high naturally occurring variance. In our analysis, we considered effects from the sample material and from pre-processing steps on VOC emissions of bacterial cultures, and also confounding effects by different inoculation days, incubation periods and varying bacterial densities for each set of samples. Nevertheless, a common set of VOCs related to the growth of MAP cultures was detected. They were assembled based on random forest classifiers, which reached high classification accuracies for their respective set of samples. Thus, we conclude that these VOCs allow to discriminate MAP-positive and negative samples despite of additional emissions from sample material. More precisely, identification of marker substances relied on three different categories of samples, (i) culture tubes containing only plain medium which were treated with either HPC or HPC/PBS, respectively, at the beginning of the cultivation period (controls), (ii) culture tubes inoculated with MAP-negative tissue or feces, and (iii) culture tubes inoculated with MAP-positive tissue or feces. The control tubes were measured concurrently to the test tubes at all time points to unveil VOCs originating from laboratory air and to elucidate the effects of sample preparation and of aging of the medium during the cultivation time of up to 20 weeks. Inclusion of control tubes enabled identification of ETBE as contaminant of laboratory air, because it was elevated in all three categories of samples only at specific dates of sample preparation. Thus, measurements of control tubes were a crucial point of our study design to identify true marker substances. If parallel measurement of control tubes will be necessary also during practical application of VOC diagnostics remains to be elucidated in further analyses.

The study design included two animal species and two matrices to exemplify the variety of settings in practical diagnostics. The MAP isolates considered in this study represent eight different genotypes of MAP type II, the most frequently observed MAP type in samples of cattle and goat (details in Supplementary Table 1). Thus, differences in VOC profiles related to different MAP strains, as reported earlier for pure bacterial cultures (14), were taken into account. Although the effects of the different MAP strains included in this study were not analyzed separately, they are expected to be negligible for the aims of the present study. For example, while cattle tissue samples exhibited the highest variance of MAP types (six different strains), they could still be classified with high accuracy, in both analyses, with a moderate number of selected VOCs.

A few VOCs related to animal species and matrix were identified. Since these effects are expected to be similar for MAP-positive and negative samples, they would not be considered informative by the variable selection procedure. Thus, the inclusion of MAP-negative diagnostic samples was crucial to identify truly MAP-related VOC emissions.

As discussed in previous studies (17, 38), random forests' ability to consider multiple compounds simultaneously makes it suitable for analysis of patterns in VOC data. Random forests have been applied before to analyze VOC patterns for other settings [see e.g., (39–43)], and the random forest-based variable selection algorithm Boruta has been deployed in other VOC studies too to select relevant compounds (44–48). In the present study, the Boruta algorithm was favored over other variable selection methods to reduce the number of VOCs to a set of potential MAP marker compounds because of its proven performance in the context of random forest classifiers (49). The number of repeated Boruta applications we chose for this analysis does not need to be as high as 30, as the algorithm is computationally expensive and a lower number would not have changed the outcome in a major way, as long as the cutoff point is similar. The averaged accuracy of the random forest classifiers is potentially biased, since Boruta was applied out of the cross-validation scheme (50). This resulted as a drawback from our decision to base our workflow on the R package caret, which enables to create reproducible workflows by using the available built-in functions, but does not yet include the Boruta algorithm.

While, random forests are straightforward to apply, as they do not require pre-processing and use only a small number of parameters, there are also some disadvantages. As random forest classifiers consist of hundreds of decision trees summarizing myriads of decision rules, it is hardly feasible to pin down the complex interplay of variables in the classifier to simple statements. Instead, we investigated variable importance values to gain insight into the results of the random forest analyses. These values are measurements of predictive power of VOCs for the particular classification task on a specific class of samples and should only be compared within the same class. Their relative rankings can be unstable (51, 52), e.g., correlated variables can produce similar importance values and also lead to underestimated importance values (53). Thus, for a cluster of variables with similar importance values, small changes in absolute importance may be associated with a large (but not meaningful) skip in ranks.

Moreover, the top compounds according to random forests' variable importance measure are not necessarily the best choice for diagnostic use. Random forests do not discriminate between VOCs with low and high concentration ranges, but screen for variables which allow to single out samples of the same class by simple decision rules. Therefore, also top VOCs with high variable importance values may not be applicable for diagnostic use, if their concentration values are too close to LOQ.

With our workflow, we analyzed the data sets two-fold: We targeted the classification procedure on (i) MAP presence and (ii) MAP growth scores, as we think of the two analyses as complementary to each other. While, the analyses targeting MAP presence are directly motivated from the study design and might profit from balanced classes, the second group of analyses targeting MAP growth scores gives additional insight into the relation of VOCs to bacterial density.

Furthermore, the different sets of samples had to be analyzed separately (goat or cattle and tissue or feces) in order to detect variations specific for the respective sample material. Comparative analyses finally showed similarities between the VOC selections for the different set of samples, especially considering MAP growth. However, since the different classes of samples were analyzed separately, quantitative differences in concentration values between these sets were not considered and consistency across the different sets of samples could not be inferred directly from our workflow, but explorative analysis showed comparable trends.

As a novelty, the present study described VOC profiles for MAP cultures derived from original sample material. Nevertheless, some VOCs of our final selection had also been included in the VOC profile for pure MAP cultures (17) and showed a consistent tendency with the previously described trend above growing MAP cultures (pentane, octane, 2-methyl-1-butanol, 3-methyl-1-butanol, acetone, 2-butanone, 2,3-butanedione, 3-pentanone, hexanal, heptanal, and benzaldehyde). In addition, further VOCs conformed to results of previous studies on VOC emissions of pure MAP cultures (15, 16), but had not been included in the VOC profile because they were described only in a single study (2-methyl-1-propanol, 1-pentanol, 1-octen-3-ol, acetaldehyde, propanal, and pentanal). However, while furans included in the published MAP core profile had consistently shown an increase in concentration above MAP, for our samples furans tended to decrease with increasing bacterial density or exhibited a varying pattern. For example, 2-pentylfuran was reported to be an important marker compound among the VOCs of the MAP core profile (17) and had been detected in high concentration ranges above MAP cultures (14), but for the present samples 2-pentylfuran showed only slight variations with respect to bacterial growth densities and did not indicate MAP presence in general. Other VOCs have been investigated before as potential MAP markers but showed differing tendencies above MAP cultures (17) (2-methylpropanal, 2-methylbutanal, 3-methylbutanal, 2-heptanone). Furthermore, some VOCs have not been described in any of the previous studies on MAP cultures (2-hexanone, 2-octanone, octanal, and nonanal).

Remarkably, the majority of MAP-positive samples without visible bacterial growth could be distinguished from negative samples by our workflow (as indicated by the confusion matrices, Supplementary Tables 6–9), apart from goat tissue samples. This underlines the potential of an early *in vitro* MAP diagnosis using VOC analysis. Compounds with a considerable difference in concentration above MAP cultures with none or scant visible bacterial growth (score 0 and 0.5) in comparison to negative samples and control vials are alcohols such as 2-methyl-1-propanol, 2-methyl-1-butanol, 3-methyl-1-butanol, and 1-octen-3-ol, aldehydes such as 2-methylbutanal, 3-methylbutanal, pentanal, hexanal, benzaldehyde, heptanal, and octanal, and furans such as 2-methylfuran, 2-ethylfuran, and 2-butylfuran (see Figure 5). Aldehydes have been described before as potential marker substances of early MAP growth (15, 16). However, these studies identified an increase in concentration of some aldehydes before MAP growth was visually apparent and a decrease with increasing bacterial density. In the present study, an increase of aldehydes could not be confirmed, which may be due to the fact that the previous study also analyzed samples after only 2 weeks of incubation.

Microorganisms can produce a wide variety of volatiles. The reasons why they produce volatiles is unclear, but several functions such as communication (54) and defense have been suggested (55). The VOCs of bacteria (pathogenic and non-pathogenic) have been studied extensively (1, 55). A variety of VOCs has been identified over mycobacterial cultures, especially *M. tuberculosis* (Mtb) and *M. bovis* strains. Most of the substances described in our study were already reported previously (56). However, the knowledge about the origin and fate of VOCs within the metabolism of MAP is still limited. Therefore, conclusions have to be drawn from other (myco)bacteria.

The majority of the substances considered important for the classification of MAP-positive samples most likely originate from carbon and fatty acid metabolism of MAP. Carbon catabolism provides the bacterial cell with energy and essential biosynthetic precursors (57). In contrast to other bacterial genera, which use catabolite repression as a regulatory mechanism to maximize growth by consuming individual carbon substrates in a preferred sequence, Mtb is able to catabolize multiple carbon sources simultaneously to augment growth (58). Consequentially, a whole range of intermediates is to be expected. The same can be assumed for MAP, although it was not demonstrated so far.

Herrold's Egg Yolk Medium, which was used for cultivation in this study, provides several carbon sources, e.g., polysaccharides of the agar, egg yolk derived cholesterol, the fatty acids oleic acid and linoleic acid, sodium pyruvate, and glycerol.

Emerging evidence, predominantly originating from studies with Mtb, suggests that fatty acids, rather than carbohydrates, might be the dominant carbon substrate utilized during infection. Fatty acids, cholesterol, glycerol as well as pyruvate are degraded to acetyl-CoA. Acetyl-CoA is further oxidized to CO_2_ by the citric acid cycle, which provides reducing equivalents for respiration-mediated ATP synthesis and essential precursors for multiple biosynthetic pathways, such as glucose-6-phosphate, acetyl-CoA and others (57). However, the actual metabolic origin of most VOCs found in the present study remains unknown.

Isoprene was produced by MAP-positive cultures and increased in concentration with increasing growth rate. It was considered highly important as indicator for the presence of MAP and for MAP growth by random forest analysis. In general, it is an important atmospheric hydrocarbon that is emitted to the atmosphere from terrestrial plants, phytoplankton sources and soil bacteria (59). Various bacterial species, both Gram-positive and Gram-negative, were found to produce it (60). One major source of isoprene is the bacterial methylerythritol phosphate pathway (61, 62), which is also utilized by Mtb for the biosynthesis of five-carbon building blocks of isoprenoids. Isoprenoids are crucial for survival of Mtb and other microorganisms. They are the parent compounds of many secondary metabolites involved in membrane function, respiratory electron transport and bacterial cell wall synthesis (63).

As far as alcohols and ketones are concerned, it is noticeable that, in the present study, substances with up to 5 carbons are rising in concentrations in the headspace of MAP-positive tubes in relation to growth, while at the same time, substances with more than 5 carbons are decreasing in concentration. It seems that the former compounds result from catabolic processes while the latter may be consumed within biosynthetic pathways. This is feasible because hydrocarbons, aliphatic alcohols and ketones presumably are formed by modification of products of the fatty acid biosynthetic pathway (55). Reverse reactions with similar intermediates take place during degradation of fatty acids through the β-oxidation pathway. Every single intermediate can potentially be the precursor of volatile compounds emitted by the bacteria (64).

For the classification of MAP and for MAP growth, 3-methyl-1-butanol, 2-methyl-1-propanol, 2-methyl-butanol and 2-heptanone were considered most important in all eight and 3-pentanone in 4 and 3 classifiers, respectively. The best discrimination was achieved by 2-methyl-propanol and 3-pentanone. McNerney et al. (65) identified seven potential markers of *M. bovis* BCG above cultures on Loewenstein-Jensen medium, a whole egg medium, among them 2-methyl-1-propanol, 2-methyl-1-butanol, 3-methyl-1-butanol, and 2-butanone, which were indicative for MAP-positive cultures in the present study. These compounds are not unique to mycobacteria. Identical methyl alcohols were identified in the headspace above fungal and other bacteria species (66, 67). This underlines that the compounds are of limited value as individual markers for detecting specific bacteria, but that their value may increase if used in combination as components of a VOC profile or “fingerprint” (65).

Methyl ketones derive from two principle metabolic pathways. First, they are formed from alkanes by alpha-oxidation with no change in the carbon skeleton. In some hydrocarbon-oxidizing bacteria of the genus *Mycobacterium*, for example, the pathway of propane metabolism involves an initial hydroxylation reaction producing isopropanol, which is oxidized subsequently to acetone (68). This may be the way of acetone formation during cultural growth of MAP. Second, methyl ketones with an odd number of carbon atoms (acetone to pentadecan-2-one) are derived from even-numbered β-keto acids by decarboxylation, and occur in many bacteria (55). 2-Butanone, 2-pentanone, 2-heptanone, and others were detected in the VOCs released by *Lactobacillus casei* (69). 2-Heptanone is produced by endophytic bacteria in plants such as *Bacillus (B.) pumilus* and *B. safensis*, and is one of several compounds with antifungal activities (70).

Mycobacteria are not only able to produce, but have also an affinity for growing on a variety of methyl ketones (71). Different rapid growing mycobacteria were shown to utilize acetone, 2-butanone, 2-pentanone, 2-tridecanone or octadecanone. The short-chain ketones supported more rapid and abundant growth than the long-chain ketones (68).

Interestingly, the concentrations of aldehydes with two to eight carbon molecules tend to decrease or are significantly lower above the MAP-positive cultures compared to negative cultures or control tubes. Different sources of these compounds have to be considered. Obviously, the culture medium is itself a source of volatiles, particularly as the autoclaving process forms several VOCs (55). Emission of aldehydes by control tubes containing HEYM was demonstrated in a previous study (15). Otherwise, aldehydes were produced by MAP cultures with a characteristic dynamic pattern, as the headspace of MAP cultures with low bacterial density contained higher concentrations of these compounds than control tubes and then cultures with higher bacterial density (15). In Mtb, aldehydes proved to be toxic metabolites of the cholesterol degradation pathway (72). In contrast to our results, the headspace of BCG cultures contained significantly more acetaldehyde than was present in the headspace of the controls (65). On the other hand, aldehydes seem to be intermediates in the biosynthesis of the lipids composing the mycobacterial cell envelope (73). Benzaldehyde and octanal, among others, are substrates of the *M. bovis* BCG alcohol dehydrogenase, which seems to play a role in this pathway (74). Furthermore, aldehydes as well as ketones could result from enzymatic or thermic degradation of mycolic acids of the mycobacterial cell wall (75, 76).

As mentioned above, furans tended to decrease above MAP-positive cultures or showed variable tendencies. Their impact on classification of cultures from diagnostic samples was not as pronounced as shown previously on pure MAP cultures (14). Similar to aldehydes, furan derivatives seem to be involved in mycobacterial cell wall formation and degradation, since mycobacterial surface glycolipids contain D-galactofuran and arabinofuranosyl-residues (77, 78). The balance between these processes may determine the kind of substances and their concentrations in the cultures. 2-Pentylfuran was suggested as marker of *Aspergillus* infection in humans (79, 80).

Dimethyl disulfide, an organosulfur compound and intermediate of methionine and cysteine degradation, was identified in varying concentrations above pure HEYM, MAP-negative and MAP-positive culture tubes. Interestingly, the lowest concentrations occurred above MAP-positive tubes with growth score 2–4. This is most likely due to consumption of the substance by replicating MAP. Recent findings that members of the *Actinobacteria* in bio-filters assimilate dimethyl disulfide contained in air emissions from livestock facilities support this assumption (81).

The results of previous (14–17) and the present study provide proof of principle that detection of MAP presence and replication is possible by analysis of VOCs in the headspace of culture tubes already at very low bacterial density and before colony growth becomes visible. Sampling was done at discrete time points during the cultivation process by pre-concentration of VOCs using different micro-extraction techniques. Volatiles were identified later *offline* by GC-MS. This enabled the detection of VOCs in very low concentrations in the ppbV—pptV range (see also Supplementary Tables 2, 3). Utilization of VOC analysis in practical diagnosis would demand a different approach. VOC emission has to be measured continuously to enable monitoring of the concentration dynamics of individual marker substances. Analytical platforms that allow online analysis of VOC emissions, such as ion mobility spectrometry (IMS), ion flow tube-mass spectrometry (SIFT-MS) or proton transfer reaction-mass spectrometry (PTR-MS), respectively, are available and could be adapted for this purpose. The incubation time of cultures minimally necessary for correct classification of samples has to be defined. A broader knowledge about the sources of the potential marker compounds and an assessment of their robustness in respect to further matrices and increased sample sizes is needed. Finally, the discriminatory performance of the adapted analysis systems compared to established diagnostic methods, in particular to direct PCR against the same samples, has to be evaluated.

## Conclusion

The present paper described VOC profiles of MAP cultures from native samples for the first time. MAP-related changes in headspace VOC composition were clearly detectable and not masked by emissions from original sample material. Most VOCs highlighted in this paper have been described for pure MAP cultures before, and some of them were included in the MAP core profile (17) showing a consistent tendency above MAP cultures in comparison to control vials. In contradiction to the published core profile, furans exhibited a decrease in concentration above MAP cultures in the present study. The reasons for this reversal remain unclear. However, the potential of VOC analysis to detect bacterial growth before colonies become visible could still be confirmed. Thus, cultural diagnosis of paratuberculosis could eventually be accelerated by monitoring VOC emissions of growing MAP bacteria. In order to develop a VOC-based diagnostic test, further validation studies are needed to increase the robustness of indicative VOC patterns for early MAP growth.

The techniques presented in this paper are not restricted to MAP, but could be applied to other bacterial cultures as well. However, influencing parameters must be taken into consideration, such as medium composition and measuring technique (pre-concentration, detection, and quantification of VOCs), which will affect the resulting VOC panel. Defined framework conditions are a prerequisite to assess a reliable VOC profile. For a first screening for putative VOC markers, the selected technique should cover a wide range of substance classes. Indicative compounds can be extracted from the full panel by random forest-based approaches, as presented here, which facilitate the consideration of multivariate VOC patterns and return a ranking of the compounds with few preconditions on the VOC data.

## Data Availability Statement

The original contributions presented in the study are included in the article/Supplementary Material, further inquiries can be directed to the corresponding author/s.

## Ethics Statement

The samples of the present study were part of the sample collection of the German National Reference Laboratory for paratuberculosis at the Friedrich-Loeffler-Institut and originated from different previous studies. One study was a slaughterhouse survey, the others were approved by the Animal Health and Welfare Unit of the Thüringer Landesamt für Verbraucherschutz (permit numbers 04-102/16 and 04-001/11).

## Author Contributions

PR, HK, JS, and WM conceived the study. AK planned the experiment and carried out sample preparations. PG carried out GC-MS measurements. PG and PT analyzed the GC-MS spectra. PM carried out genotype analyses of the MAP-isolates. Data analysis was carried out by PV and EK, while the data analysis strategy was conceived by PV, EK, and HK. PV created the workflow and the R Shiny app. PV, EK, and HK drafted the manuscript. All authors discussed the results and commented on the manuscript. All authors read and approved the final manuscript.

## Conflict of Interest

The authors declare that the research was conducted in the absence of any commercial or financial relationships that could be construed as a potential conflict of interest.
