# Supplementary material for: Detection of Mycobacterium avium ssp. paratuberculosis in Cultures From Fecal and Tissue Samples Using VOC Analysis and Machine Learning Tools
Source: Front Vet Sci. 2021 Feb 3;8:620327. doi: 10.3389/fvets.2021.620327 (PMC7887282; doi:10.3389/fvets.2021.620327)
Supplement: Supplementary file 1 [file Data_Sheet_1.pdf]

Supplementary Material

1 FIGURES

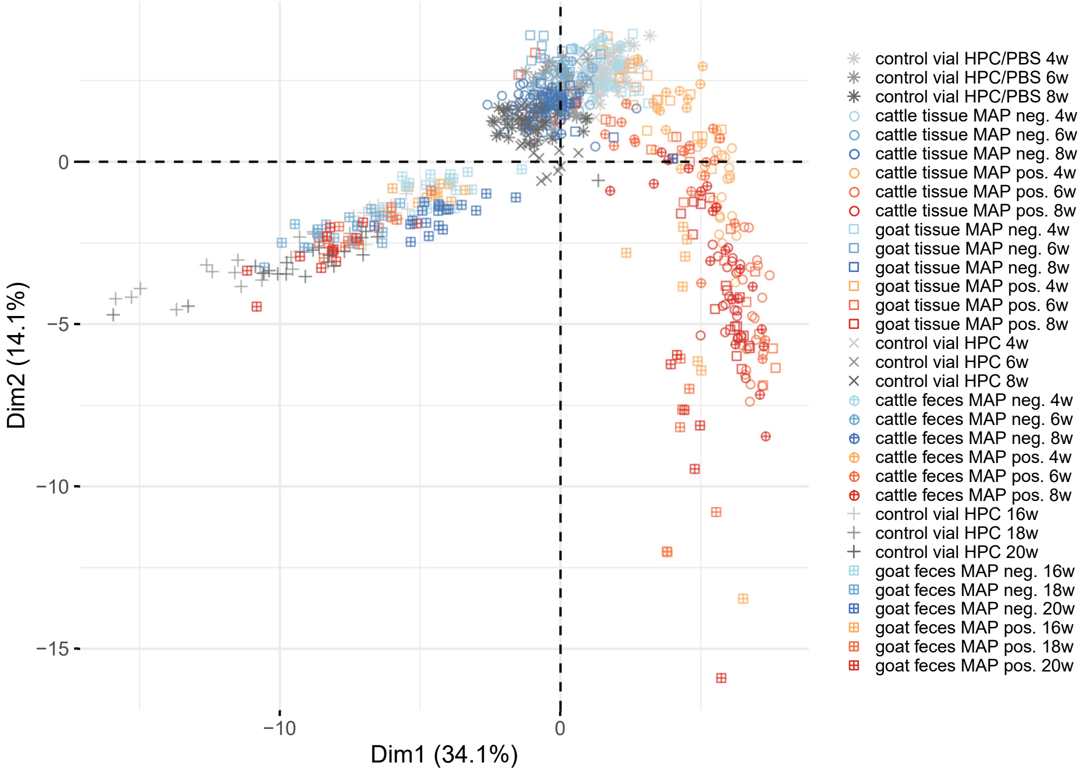

Figure S1. Differentiation of MAP-positive and negative samples by VOC emissions based on principal component analysis (PCA). Symbols indicate species (cattle or goat), matrix (feces or tissue), incubation period, and MAP presence (grey: control vials, blue: MAP-negative samples, red: MAP-positive samples).

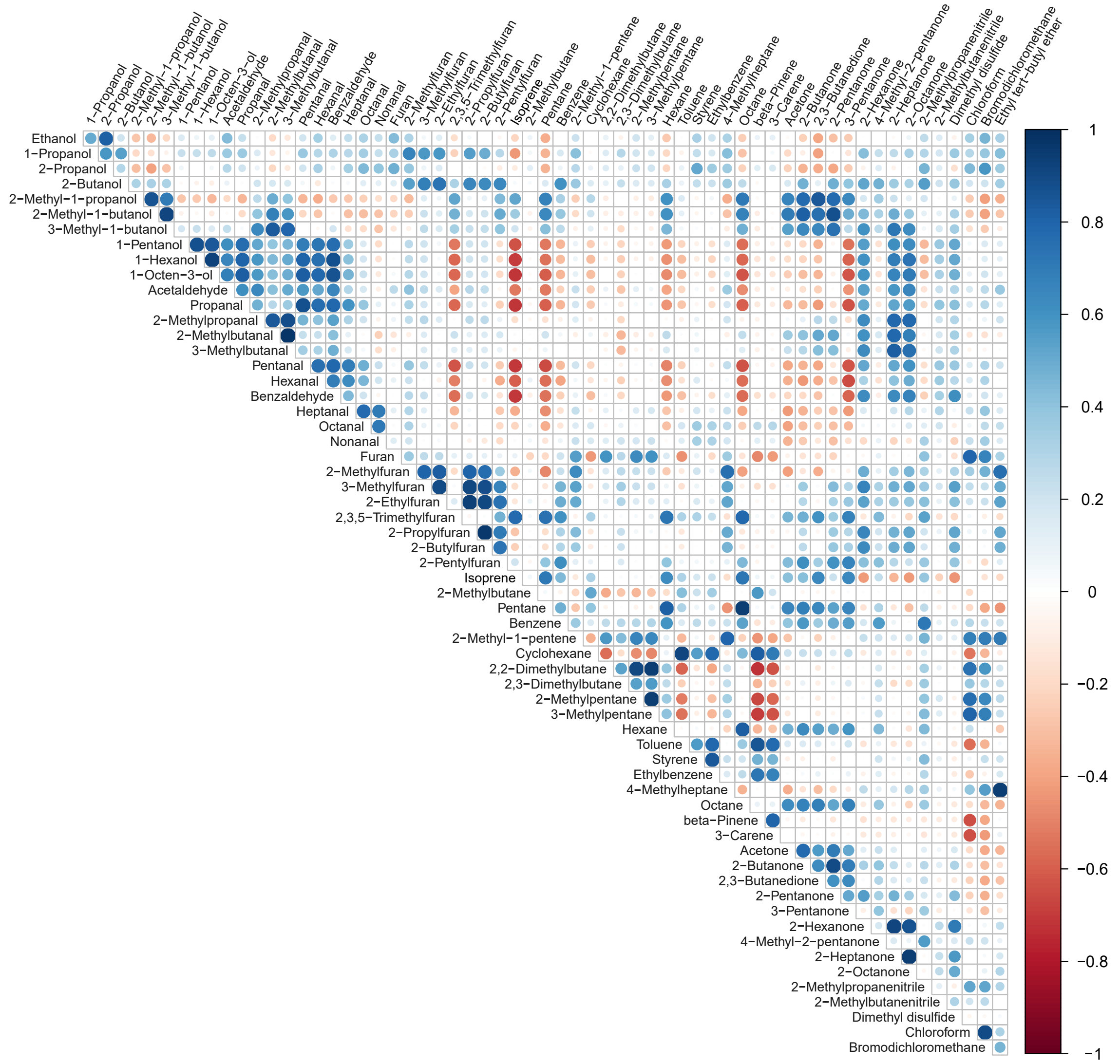

Figure S2. Correlation heat map using Spearman's rank correlation coefficient for VOCs in headspace above MAP bacterial cultures with visible growth (both from fecal and tissue samples, cattle and goat).

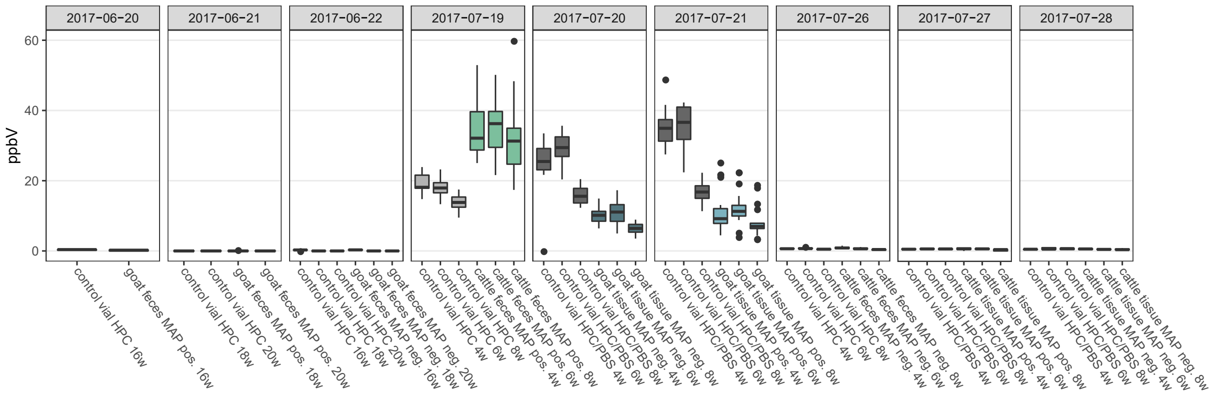

Figure S3. Concentration levels of ethyl tert-butyl ether show a systematic bias depending on the date of inoculation. This compound most likely originates from exogenous sources.

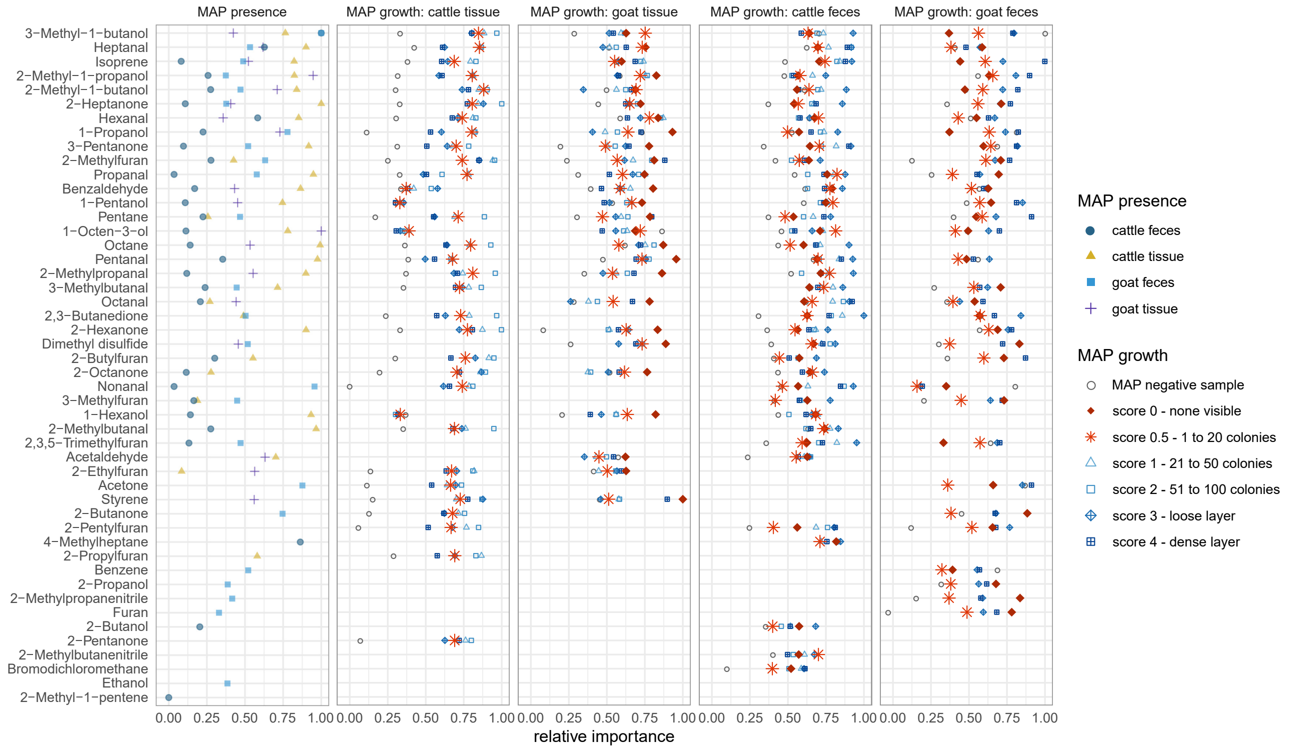

Figure S4. Variable importance values for all random forests analyses. Relative importance values are derived by dividing by the maximum importance value per analysis. Compounds on the y-axis are tentatively ordered by selection frequency and mean importance. Compounds that were not selected in any analysis are not shown. Variable importance values for early phases of bacterial growth are highlighted in red.

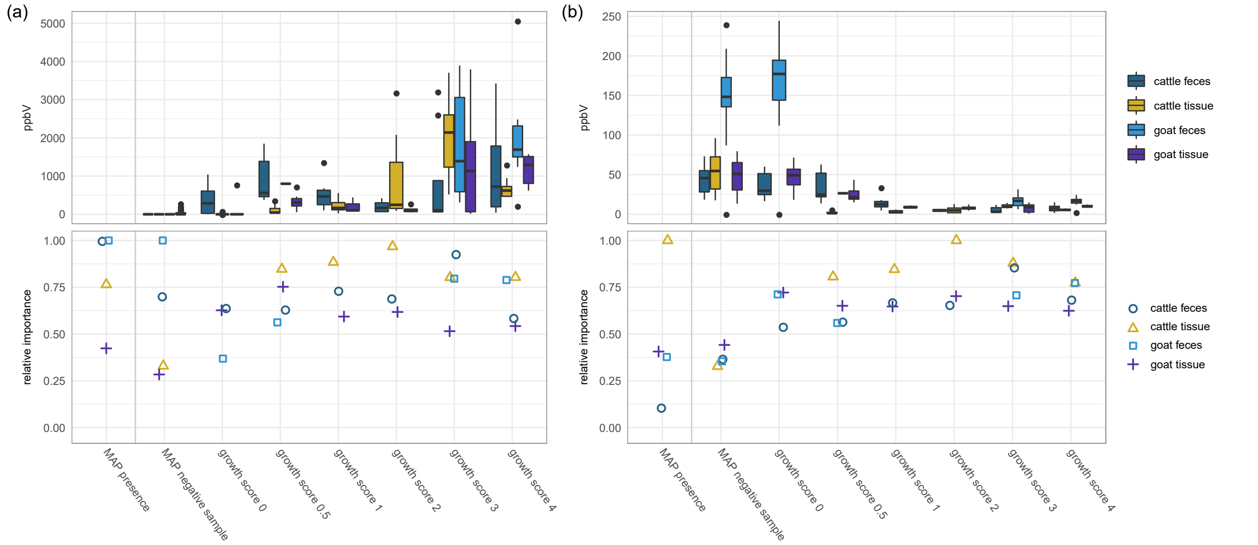

Figure S5. Comparison of concentration values (top) and relative importance values (bottom) per growth score for (a) 3-methyl-1-butanol and (b) 2-heptanone. Changes in relative importance between growth scores do not necessarily translate into changes in concentration values and vice versa.

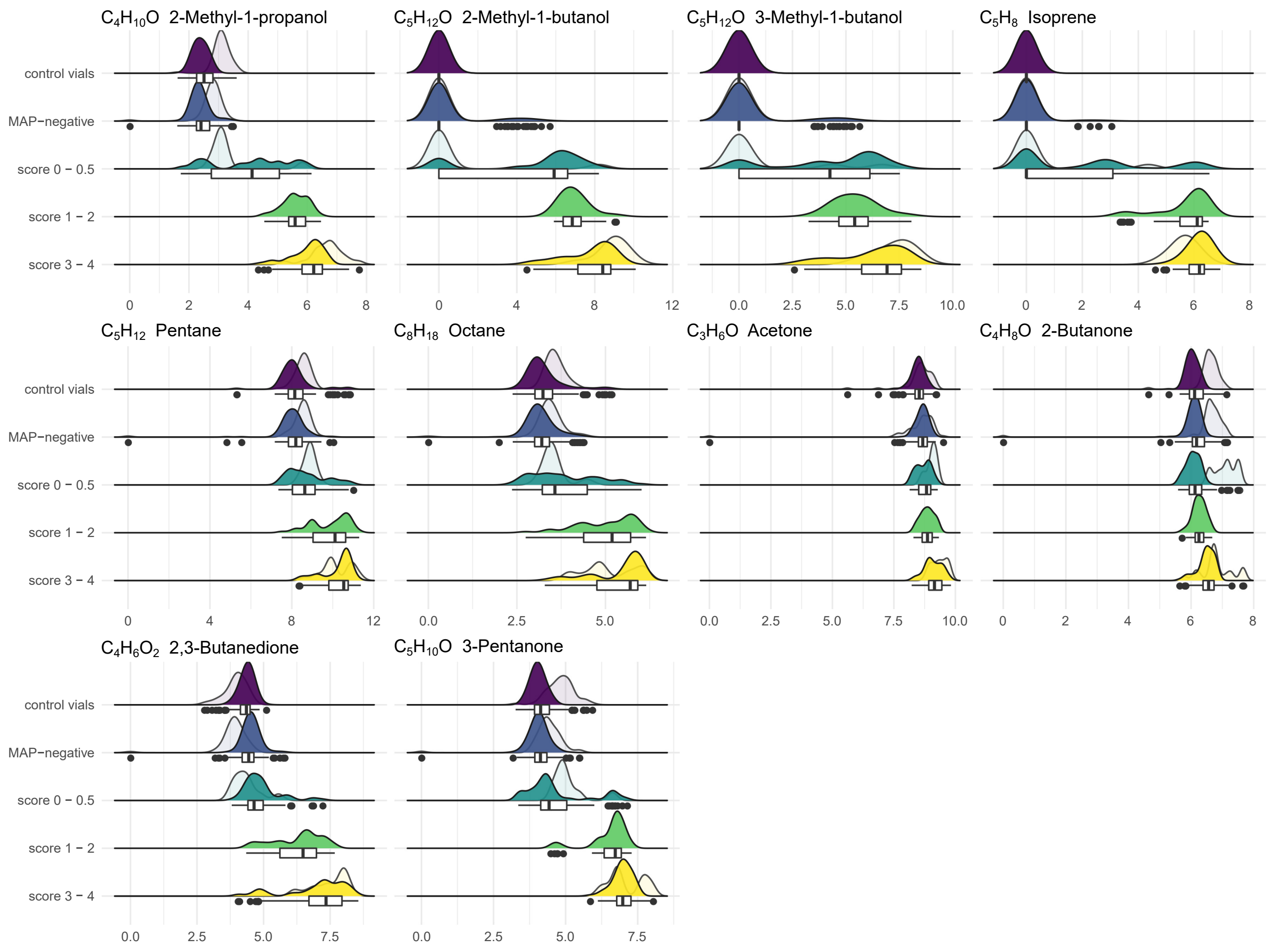

Figure S6. Overview of VOCs with an increase in concentration in relation to MAP status and growth scores which where selected in at least two settings by our workflow for the analysis targeting MAP presence or MAP growth scores. The figure shows the distribution of log(1+x)-transformed concentration values by smoothed histograms (filled: incubation period of four, six and eight weeks, transparent: incubation period of 16, 18 and 20 weeks) and by box-whisker plots. Goat feces samples that did not show any bacterial growth after 20 weeks of incubation were excluded for this visualization. For details on the scoring scheme for MAP growth see legend of Figure S4.

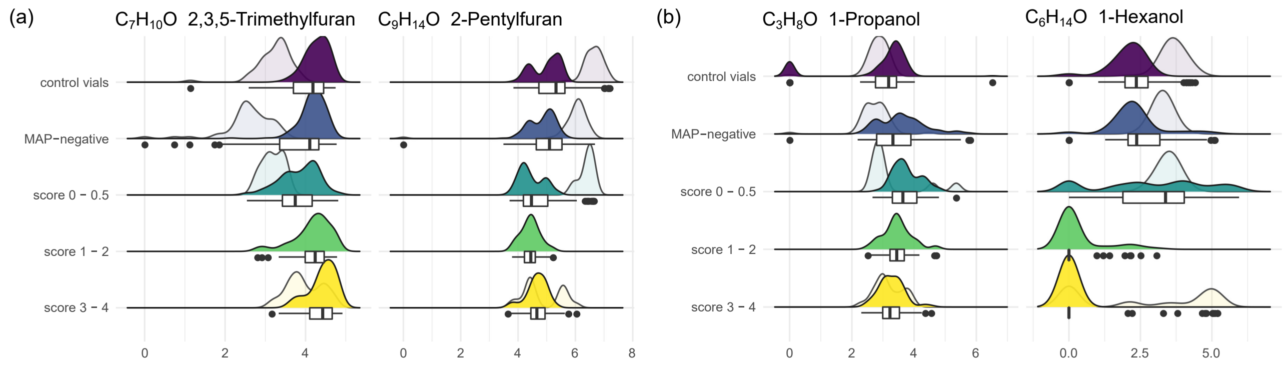

Figure S7. VOCs with varying patterns in relation to MAP growth: (a) VOCs with a decrease for early bacterial growth and increase for higher bacterial densities, (b) VOCs with an increase for early bacterial growth and decrease for higher bacterial densities. For details see Figure S6.

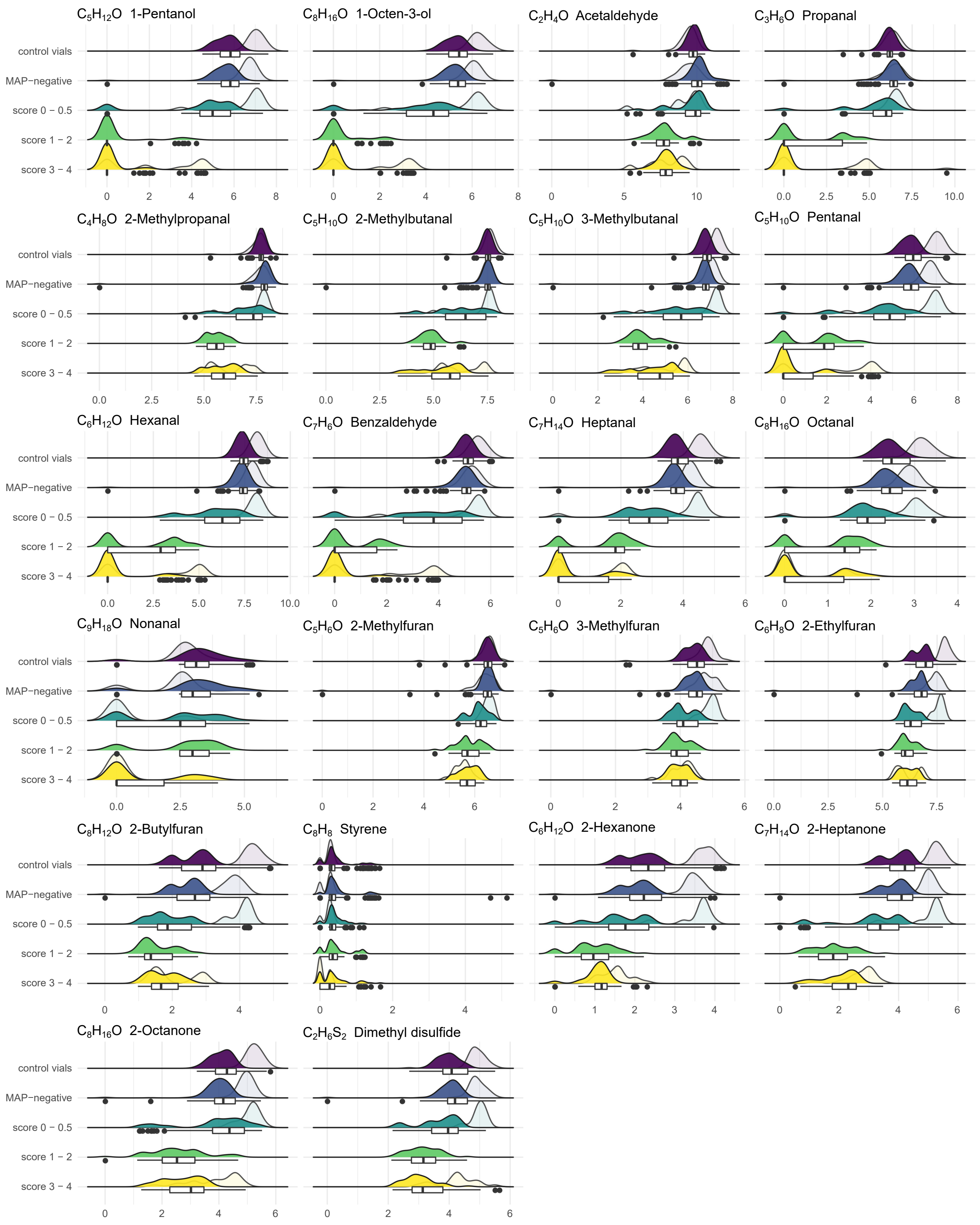

Figure S8. VOCs with a decrease in concentration in relation to MAP growth. For details see Figure S6.

2 TABLES

Table S1. Genotypes of the MAP-isolates culturally recovered from the different samples. Genotypes were derived from the combined results of three different methods: MIRU-VNTR typing (Thibault et al., 2007) and SSR sequencing (Amonsin et al., 2004), modified according to (Fritsch et al., 2012); and SNP-based assay revealing the Phylogenetic Clade (Leão et al., 2016)[1].

Matrix	Species	Herd	Animal No.	Sample No.	Genotype VNTR/SSR	Phylogenetic Clade	Combined Genotype
Feces	Cattle	A	1	1	2/744	4	I
			2	2			
			3	3			
		B	4	4	2/744	2	II
			5	5			
		C	6	6	19/744	6	III
			7	7			
		D	8	8	2/744	4	I
			9	9			
			10	10			
Feces	Goat	E	1	1	No growth	n.d.	
			2	2			
			3	3			
			4	4			
			1	5			
		F	5	6	3/744	4	IV
			5	7			
		G	6	8	INMV 11'/744	3	V
			7	9			
			6	10			
Tissue	Cattle	H	1	1	2/745	4	VI
			1	2			
		J	2	3	2/744	4	I
			2	4			
		K	3	5	30'/744	Subgroup B	VII
			3	6			
		L	4	7	19/744	6	III
			4	8			
		M	5	9	3/744	4	IV
			5	10	3 and 5/744	4 and 2	Mix (IV and VIII)
Tissue	Goat	N	1	1	19/744	6	III
			1	2			
			2	3			
			2	4			
			3	5			
			3	6			
			4	7			
			4	8			
			5	9			
			5	10			

[1] References:

Amonsin A, Li LL, Zhang Q, Bannantine JP, Motiwala AS, Sreevatsan S, Kapur V. Multilocus short sequence repeat sequencing approach for differentiating among *Mycobacterium avium* subsp. *paratuberculosis* strains. *J Clin Microbiol* (2004) 42:1694-1702. doi: 10.1128/JCM.42.4.1694-1702.2004

Fritsch I, Luyven G, Köhler H, Lutz W, Möbius P. Suspicion of *Mycobacterium avium* subsp. *paratuberculosis* transmission between cattle and wild-living red deer (*Cervus elaphus*) by multitarget genotyping. *Appl Environ Microbiol* (2012) 78:1132-1139. doi:10.1128/AEM.06812-11

Leão C, Goldstone RJ, Bryant J, McLuckie J, Inácio J, Smith DGE, Stevenson K. Novel single nucleotide polymorphism-based assay for genotyping *Mycobacterium avium* subsp. *paratuberculosis*. *J Clin Microbiol* (2016) 54:556-564. doi: 10.1128/JCM.01958-15

Thibault VC, Grayon M, Boschiroli ML, Hubbans C, Overduin P, Stevenson K, Gutierrez MC, Supply P, Biet F. New variable-number tandem-repeat markers for typing *Mycobacterium avium* subsp. *paratuberculosis* and *M. avium* strains: Comparison with IS900 and IS1245 restriction fragment length polymorphism typing. *J Clin Microbiol* (2007) 45:2404-2410. doi: 10.1128/JCM.00476-07

Table S2. Calibration data and limits of detection (LOD) / limits of quantification (LOQ) for reference substances.

Compound	Retention time (min)	Quant. ion (Th)	Slope (counts / ppbV)	R^2	LOD (ppbV)	LOQ (ppbV)
Ethanol	6.638	45	67.68	0.974	25.995	50.825
1-Propanol	9.655	42	83.95	0.996	2.391	4.306
2-Propanol	7.712	45	668.34	0.994	3.528	8.615
2-Butanol	11.072	45	812.24	0.984	0.235	0.443
2-Methyl-1-propanol	12.105	43	604.50	0.990	0.539	1.157
2-Methyl-1-butanol	15.375	57	984.27	0.986	0.265	0.572
3-Methyl-1-butanol	15.445	43	434.76	0.988	0.679	1.381
1-Pentanol	16.437	42	397.64	0.988	1.001	2.178
1-Hexanol	19.464	56	441.73	0.997	0.424	0.828
1-Octen-3-ol	22.268	57	670.50	0.999	0.774	1.693
Acetaldehyde	4.604	44	31.29	0.977	47.348	159.269
Propanal	7.236	58	101.17	0.946	12.077	24.970
2-Methylpropanal	9.230	43	212.93	1.000	2.027	3.840
2-Methylbutanal	12.854	41	503.91	0.999	1.829	3.660
3-Methylbutanal	12.601	41	2379.70	0.978	0.272	0.480
Pentanal	13.978	44	672.61	0.993	2.838	5.097
Hexanal	17.328	44	611.00	0.999	7.019	15.994
Benzaldehyde	22.774	105	1470.50	0.999	1.606	3.365
Heptanal	20.456	70	420.29	0.999	1.780	3.510
Octanal	22.845	41	715.63	0.994	1.627	2.558
Nonanal	25.184	57	1020.50	0.975	4.534	10.379
Furan	6.710	39	175.35	0.953	3.820	8.080
2-Methylfuran	10.070	82	727.08	0.994	0.391	0.868
3-Methylfuran	10.354	82	648.46	0.995	0.269	0.552
2-Ethylfuran	13.310	81	1702.50	0.996	0.181	0.385
2,3,5-Trimethylfuran	16.711	110	1790.50	0.995	0.102	0.214
2-Propylfuran	15.942	81	3235.00	0.997	0.511	0.812
2-Butylfuran	19.434	81	4691.00	0.996	0.178	0.375
2-Pentylfuran	22.094	81	3907.30	0.998	0.434	0.942
Isoprene	6.730	67	351.75	0.992	1.813	3.679
2-Methylbutane	5.464	41	127.93	0.977	8.742	16.296
Pentane	6.061	43	127.88	0.963	5.170	12.040
Benzene	12.277	78	2299.30	1.000	0.753	1.338
2-Methyl-1-pentene	8.805	56	8712.00	0.976	0.024	0.050
Cyclohexane	11.568	56	2813.60	0.999	0.080	0.178
2,2-Dimethylbutane	7.043	57	206.49	0.991	1.060	2.423
2,3-Dimethylbutane	7.975	42	6559.30	0.999	0.066	0.151
2-Methylpentane	8.066	43	328.39	0.996	1.151	2.711
3-Methylpentane	8.582	57	449.78	0.983	0.445	0.961
Hexane	9.119	57	3243.00	0.989	0.064	0.134
Toluene	15.527	91	2539.81	0.984	0.541	0.967
Styrene	19.991	104	8212.60	1.000	0.131	0.266
Ethylbenzene	18.867	91	13 848.00	1.000	0.101	0.134
4-Methylheptane	14.696	43	8566.00	0.996	0.047	0.106
Octane	15.516	43	4977.00	1.000	0.245	0.555
beta-Pinene	21.742	93	8748.00	0.990	0.038	0.085
3-Carene	22.185	93	11 123.00	0.989	0.426	0.977
Acetone	7.438	43	378.84	0.988	15.872	29.106
2-Butanone	10.829	43	1171.30	1.000	1.553	3.130
2,3-Butanedione	10.414	43	1251.00	0.999	1.701	3.441
2-Pentanone	13.633	43	2188.80	0.999	0.527	1.135
3-Pentanone	13.897	57	2205.00	0.997	0.360	0.777
2-Hexanone	16.809	58	3176.00	0.954	0.417	0.791
4-Methyl-2-pentanone	15.395	43	7872.00	0.976	0.066	0.120
2-Heptanone	20.234	43	3967.20	0.981	0.303	0.649
2-Octanone	22.633	43	3466.00	0.993	0.314	0.602
2-Methylpropanenitrile	12.439	42	1773.80	0.992	0.161	0.334
2-Methylbutanenitrile	15.567	55	4298.00	0.992	0.169	0.373
Dimethyl disulfide	15.304	94	1267.30	0.748	0.146	0.291
Chloroform	11.325	83	711.41	0.985	0.258	0.478
Bromodichloromethane	14.352	83	9091.10	0.999	0.018	0.037
Ethyl tert-butyl ether	10.131	59	2383.30	0.990	0.065	0.132

Table S3. Concentration ranges for control vials, MAP-negative samples and MAP-positive samples (1st qu.: first quartile, 3rd qu.: third quartile, prop. 0: proportion of zeros).

	control vials				MAP-negative samples				MAP-positive samples			
Compound	1st qu. (ppbV)	median (ppbV)	3rd qu. (ppbV)	prop. 0	1st qu. (ppbV)	median (ppbV)	3rd qu. (ppbV)	prop. 0	1st qu. (ppbV)	median (ppbV)	3rd qu. (ppbV)	prop. 0
Ethanol	426 825.98	511 997.54	611 082.18	0.000	476 032.42	593 457.14	668 536.29	0.004	466 247.54	543 632.25	626 150.88	0.004
1-Propanol	14.81	23.11	30.03	0.134	15.41	26.68	48.27	0.004	20.07	29.50	39.56	0.008
2-Propanol	2372.69	2961.30	3409.11	0.000	2613.36	3351.61	3748.57	0.004	2362.29	2942.94	3428.90	0.004
2-Butanol	23.32	26.87	31.19	0.000	24.57	27.37	31.57	0.004	19.50	23.00	27.14	0.004
2-Methyl-1-propanol	8.58	11.30	15.63	0.000	8.53	10.11	13.89	0.004	51.85	247.71	469.47	0.004
2-Methyl-1-butanol	0.00	0.00	0.00	1.000	0.00	0.00	0.00	0.917	154.66	865.77	3580.45	0.218
3-Methyl-1-butanol	0.00	0.00	0.00	1.000	0.00	0.00	0.00	0.917	26.16	284.64	821.48	0.172
1-Pentanol	208.57	340.04	529.96	0.000	218.93	336.76	506.81	0.004	0.00	0.00	151.09	0.542
1-Hexanol	6.11	9.56	14.87	0.034	6.90	9.69	23.05	0.029	0.00	0.00	25.89	0.567
1-Octen-3-ol	145.18	228.72	325.48	0.000	150.21	218.58	300.76	0.004	0.00	0.00	79.42	0.559
Acetaldehyde	12 753.98	16 794.85	22 389.49	0.000	15 290.76	23 056.09	30 712.32	0.004	1962.41	3761.34	16 780.86	0.004
Propanal	419.26	489.86	571.28	0.000	492.00	607.23	762.54	0.004	0.00	26.17	409.92	0.487
2-Methyl-propanal	2049.35	2252.36	2526.84	0.000	2274.08	2638.78	3060.34	0.004	237.62	544.26	1735.74	0.004
2-Methyl-butanal	1644.77	1863.20	2076.50	0.000	1603.82	1830.31	2033.58	0.004	133.46	337.64	1217.43	0.004
3-Methyl-butanal	779.05	938.60	1124.27	0.000	770.81	883.87	1019.63	0.004	44.38	140.65	381.18	0.004
Pentanal	265.24	380.05	547.79	0.000	246.02	345.03	487.23	0.004	0.00	6.86	131.70	0.395
Hexanal	1386.80	1711.22	2169.84	0.000	1377.64	1641.99	2088.30	0.004	0.00	35.75	549.29	0.403
Benzaldehyde	139.43	166.43	205.31	0.000	132.24	159.77	186.19	0.004	0.00	3.44	50.44	0.496
Heptanal	36.44	44.67	62.72	0.000	35.64	42.20	55.50	0.004	0.00	5.97	17.76	0.357
Octanal	8.65	10.78	17.09	0.000	8.37	10.34	14.04	0.004	0.00	3.53	6.52	0.345
Nonanal	13.44	21.46	36.06	0.067	11.71	18.64	38.14	0.133	0.00	0.00	18.74	0.529
Furan	193.54	238.59	323.72	0.004	205.47	306.93	399.93	0.004	221.86	313.60	405.21	0.004
2-Methyl-furan	581.54	673.63	787.88	0.000	566.05	668.28	775.44	0.004	268.21	412.32	567.91	0.004
3-Methyl-furan	67.95	90.71	113.87	0.000	70.01	90.44	107.12	0.004	43.40	57.10	83.95	0.004
2-Ethylfuran	685.69	1070.65	1477.03	0.000	633.86	887.14	1143.16	0.004	370.06	510.78	842.10	0.004
2,3,5-Tri-methylfuran	39.39	64.61	84.60	0.000	27.68	59.68	74.94	0.004	32.68	61.71	86.46	0.004
2-Propylfuran	129.15	216.99	330.15	0.000	110.91	165.26	273.44	0.004	44.23	83.04	165.49	0.004
2-Butylfuran	8.77	17.08	25.81	0.000	7.49	13.45	21.46	0.004	2.92	5.23	11.56	0.004
2-Pentylfuran	111.91	204.12	278.86	0.000	102.06	162.38	251.66	0.004	75.13	102.10	156.64	0.004
Isoprene	0.00	0.00	0.00	1.000	0.00	0.00	0.00	0.975	0.00	298.45	506.95	0.269
2-Methyl-butane	80.67	674.95	1300.95	0.017	79.17	696.21	1475.10	0.017	142.51	726.46	1268.63	0.008
Pentane	2501.68	3442.26	5116.33	0.000	2523.23	3619.44	4926.84	0.004	6058.40	14 880.44	39 542.22	0.004
Benzene	116.56	134.38	154.94	0.000	109.18	124.17	145.59	0.004	109.26	130.77	146.64	0.004
2-Methyl-1-pentene	2.36	3.15	4.29	0.000	2.09	2.75	3.91	0.004	2.00	3.16	4.59	0.004
Cyclohexane	0.39	0.53	0.91	0.004	0.50	0.73	1.19	0.004	0.55	0.76	1.29	0.013
2,2-Dimethyl-butane	12.96	30.95	65.89	0.004	11.61	39.11	74.58	0.004	9.78	30.94	84.56	0.034
2,3-Dimethyl-butane	0.76	1.50	3.88	0.025	0.98	2.12	3.58	0.008	2.10	5.00	10.24	0.004
2-Methyl-pentane	144.83	228.78	426.46	0.000	154.70	277.60	412.87	0.004	165.45	293.43	567.25	0.004
3-Methyl-pentane	52.81	85.09	137.89	0.000	47.42	93.52	135.75	0.004	42.42	80.28	154.15	0.004
Hexane	15.31	19.95	29.59	0.000	13.32	19.42	29.31	0.004	19.41	33.25	52.56	0.004
Toluene	3.27	4.11	6.38	0.004	3.09	3.90	6.74	0.004	3.51	4.88	10.42	0.013
Styrene	0.29	0.37	0.51	0.139	0.29	0.38	0.54	0.112	0.27	0.34	0.52	0.227
Ethylbenzene	0.00	0.19	0.30	0.311	0.00	0.18	0.32	0.279	0.00	0.19	0.36	0.298
4-Methyl-heptane	0.40	0.78	1.17	0.008	0.40	0.65	1.18	0.004	0.42	1.04	2.61	0.008
Octane	19.29	24.45	32.33	0.000	19.11	23.61	29.56	0.004	32.45	105.35	299.13	0.004

Table S3. Continued from previous page.

	control vials				MAP-negative samples				MAP-positive samples			
Compound	1st qu. (ppbV)	median (ppbV)	3rd qu. (ppbV)	prop. 0	1st qu. (ppbV)	median (ppbV)	3rd qu. (ppbV)	prop. 0	1st qu. (ppbV)	median (ppbV)	3rd qu. (ppbV)	prop. 0
beta-Pinene	0.00	0.21	0.76	0.403	0.00	0.21	0.87	0.417	0.00	0.22	1.04	0.391
3-Carene	0.00	0.00	0.00	0.840	0.00	0.00	0.00	0.787	0.00	0.00	0.76	0.748
Acetone	4276.60	5070.16	5949.99	0.000	4904.40	5849.55	7021.56	0.004	6291.98	7951.34	10 237.14	0.004
2-Butanone	381.87	450.34	592.64	0.000	419.64	479.85	617.20	0.004	461.45	604.15	823.76	0.004
2,3-Butanedione	61.26	76.84	88.45	0.000	65.45	83.94	102.13	0.004	95.75	355.41	1379.62	0.004
2-Pentanone	120.31	184.87	276.68	0.000	132.45	182.98	261.14	0.004	137.05	224.22	337.71	0.004
3-Pentanone	49.48	61.41	83.52	0.000	50.04	61.03	76.51	0.004	101.92	757.97	1060.26	0.004
2-Hexanone	5.70	9.35	14.56	0.000	5.50	8.25	13.40	0.004	1.71	2.70	6.65	0.063
4-Methyl-2-pentanone	4.00	4.45	4.96	0.000	4.11	4.48	4.86	0.004	4.01	4.73	5.34	0.004
2-Heptanone	39.57	66.48	92.94	0.000	36.37	59.28	86.86	0.004	5.03	11.69	31.54	0.008
2-Octanone	47.43	71.43	99.35	0.000	45.94	62.69	95.96	0.004	9.61	27.94	98.04	0.008
2-Methyl-propanenitrile	9.59	11.05	12.29	0.000	10.36	11.67	13.14	0.004	8.71	11.42	13.18	0.004
2-Methyl-butanenitrile	7.11	8.36	9.61	0.000	7.31	8.82	9.71	0.004	6.88	8.83	12.35	0.088
Dimethyl disulfide	43.37	58.19	99.48	0.000	50.81	65.12	98.28	0.004	17.45	30.79	71.91	0.004
Chloroform	246.22	339.78	442.56	0.000	268.61	383.06	513.23	0.004	246.85	363.42	481.88	0.004
Bromodi-chloromethane	1.48	2.23	2.82	0.004	1.66	2.41	2.95	0.012	1.46	2.08	2.68	0.004
Ethyl tert-butyl ether	0.33	0.64	17.45	0.176	0.26	0.52	2.00	0.188	0.25	0.96	21.54	0.227

Table S4. Statistical analyses for control vials included an assessment of the effect of the prolonged incubation period and a tentative screening for VOCs originating from exogenous sources. The screening was carried out in two steps: First, we assessed whether VOC concentration in the headspace volume of control vials differed significantly between different days of inoculation using Kruskal-Wallis tests with Bonferroni p-value correction. Such significant variations in VOC concentration can also occur naturally and we only considered them problematic, if the VOC concentration in the headspace volume of control vials did not significantly differ from the VOC concentration in the headspace volume of all test tubes (both goat and cattle, both tissue and feces, both MAP-positive and MAP-negative). Thus, this was checked in the second step using two-sided Mann-Whitney-U tests with Bonferroni correction. These two steps were performed separately for the set of control vials that had been incubated for four to eight weeks and for those that had been incubated for 16 to 20 weeks. Finally, we excluded VOCs showing significant differences between inoculation days (Kruskal-Wallis test), but not between control vials and test tubes (Mann-Whitney U test) for both incubation periods. These criteria applied only for ethyl tert-butyl ether. Concentrations of 2-methyl-1-butanol, 3-methyl-1-butanol and isoprene did not exceed the limit of quantification for any of the control vials. Therefore, statistical tests could not be applied to compare different sets of control vials for these three compounds. (K.-W.: Kruskal-Wallis, d.f.: degrees of freedom, adj.: adjusted (Bonferroni correction, $n = 62$), inc./dec.: increase (↑) or decrease (↓) in concentration with extended incubation period (reported only for significant differences), n.a.: not applicable).

	Difference between incubation periods (4-6 and 16-20 weeks)			Differences between inoculation days				Difference between control vials and actual samples			
				4-6 weeks		16-20 weeks		4-6 weeks		16-20 weeks	
Compound	Mann-Whitney U statistic	p-value (adj.)	inc./dec.	K.-W. rank sum statistic (5 d.f.)	p-value (adj.)	K.-W. rank sum statistic (2 d.f.)	p-value (adj.)	Mann-Whitney U statistic	p-value (adj.)	Mann-Whitney U statistic	p-value (adj.)
Ethanol	1515	**2.7e-14**	↓	7.37	1	1.23	1	24 961	**0.0012**	2718	1
1-Propanol	2764.5	**4.1e-06**	↓	110.64	**1.8e-20**	20.99	**0.0016**	22 082	**1.6e-07**	3082.5	1
2-Propanol	348	**7.2e-25**	↓	27.93	**0.0022**	0.81	1	26 405	**0.039**	2599	0.39
2-Butanol	8286	**1.1e-09**	↑	18.26	0.16	1.89	1	34 897	1	4696	**0.01**
2-Methyl-1-propanol	10 243	**1.9e-26**	↑	24	**0.013**	5.91	1	18 978	**4.4e-13**	4685	**0.011**
2-Methyl-1-butanol	5220	n.a.		n.a.	n.a.	n.a.	n.a.	15 570	**4.4e-29**	2871	**0.046**
3-Methyl-1-butanol	5220	n.a.		n.a.	n.a.	n.a.	n.a.	15 390	**1.6e-29**	2610	**0.0022**
1-Pentanol	10 326	**2.5e-27**	↑	2.51	1	12.61	0.11	46 400	**3.2e-15**	5536	**1.1e-08**
1-Hexanol	10 343	**1.6e-27**	↑	6.14	1	6.28	1	39 276	**0.0018**	5334.5	**5.4e-07**
1-Octen-3-ol	10 305	**4.2e-27**	↑	1.24	1	9.96	0.41	48 332	**9.9e-20**	5059	**6.0e-05**

Table S4. Continued from previous page.

	Difference between incubation periods (4-6 and 16-20 weeks)			Differences between inoculation days				Difference between control vials and actual samples			
				4-6 weeks		16-20 weeks		4-6 weeks		16-20 weeks	
Compound	Mann-Whitney U statistic	p-value (adj.)	inc./dec.	K.-W. rank sum statistic (5 d.f.)	p-value (adj.)	K.-W. rank sum statistic (2 d.f.)	p-value (adj.)	Mann-Whitney U statistic	p-value (adj.)	Mann-Whitney U statistic	p-value (adj.)
Acetaldehyde	2902	**2.2e-05**	↓	9.44	1	3.38	1	34 757	1	3481	1
Propanal	7200	**8.4e-04**	↑	18.87	0.12	3.46	1	37 502	0.11	3039	1
2-Methylpropanal	4145	1		2.84	1	3.09	1	37 625	0.092	3083	1
2-Methylbutanal	7513	**2.9e-05**	↑	7.81	1	4.78	1	46 185	**1.4e-14**	4853	**0.0013**
3-Methylbutanal	10 211	**4.2e-26**	↑	7.78	1	13.83	0.059	49 186	**1.3e-21**	5466	**4.5e-08**
Pentanal	10 298	**5.0e-27**	↑	5.36	1	12.75	0.1	50 023	**6.2e-24**	5720	**2.3e-10**
Hexanal	10 260	**1.3e-26**	↑	9.07	1	15.99	**0.02**	49 021	**2.7e-21**	5648	**1.1e-09**
Benzaldehyde	9893	**7.2e-23**	↑	8.37	1	13.94	0.056	48 765	**9.8e-21**	5393	**1.8e-07**
Heptanal	10 253	**1.5e-26**	↑	15.74	0.45	12.79	0.099	49 139.5	**1.5e-21**	6064	**6.6e-14**
Octanal	10 269	**1.0e-26**	↑	23.72	**0.014**	7.27	1	47 831	**2.6e-18**	5938	**1.4e-12**
Nonanal	2551	**2.8e-07**	↓	43.83	**1.5e-06**	13.61	0.066	39 561	**9.1e-04**	5745.5	**3.8e-11**
Furan	5702	1		6.92	1	5.36	1	21 781.5	**5.3e-08**	3157	1
2-Methylfuran	5767	1		2.9	1	2.2	1	44 500	**3.3e-11**	4568	**0.046**
3-Methylfuran	9267	**4.2e-17**	↑	5.56	1	6.32	1	42 548	**7.9e-08**	3993	1
2-Ethylfuran	10 263	**1.2e-26**	↑	5.45	1	12.57	0.11	47 986	**1.2e-18**	6451	**1.9e-18**
2,3,5-Trimethylfuran	100	**1.8e-27**	↓	2.59	1	1.11	1	36 314	1	4897	**6.8e-04**
2-Propylfuran	10 399	**4.1e-28**	↑	6.84	1	14.77	**0.037**	47 622	**8.6e-18**	6447	**2.1e-18**
2-Butylfuran	10 393	**4.7e-28**	↑	1.07	1	15.56	**0.025**	48 780.5	**1.3e-20**	6552	**9.5e-20**
2-Pentylfuran	10 439	**1.5e-28**	↑	4.45	1	17.34	**0.01**	43 677	**1.0e-09**	6361	**2.4e-17**
Isoprene	5220	n.a.		n.a.	n.a.	n.a.	n.a.	17 910	**1.1e-23**	2871	**0.046**
2-Methylbutane	4160	1		6.28	1	2.64	1	31 898	1	3040	1
Pentane	8264	**1.5e-09**	↑	28.76	**0.0015**	21.71	**0.0011**	20 181	**9.2e-11**	2546	0.23
Benzene	5464	1		6.49	1	6.07	1	37 359	0.16	4237	1
2-Methyl-1-pentene	4490	1		16.37	0.35	2.92	1	33 241.5	1	4863	**0.0011**
Cyclohexane	3345.5	**0.0023**	↓	22.03	**0.03**	1.2	1	24 508.5	**3.6e-04**	1711	**2.5e-06**
2,2-Dimethylbutane	4209	1		6.67	1	0.14	1	30 954	1	3793.5	1
2,3-Dimethylbutane	4051.5	0.61		8.95	1	0	1	21 667.5	**3.4e-08**	2893	1
2-Methylpentane	3024	**8.7e-05**	↓	7.87	1	0.15	1	28 481	1	3013	1
3-Methylpentane	3331	**0.002**	↓	5.44	1	0	1	31 458	1	3848	1
Hexane	2728	**2.7e-06**	↓	9.69	1	0.64	1	24 485	**3.4e-04**	3625	1
Toluene	3024	**8.7e-05**	↓	5.96	1	0.21	1	29 455	1	3700	1
Styrene	3480	**0.0079**	↓	7.9	1	3.27	1	31 656	1	4208	1
Ethylbenzene	2263	**2.7e-09**	↓	15.36	0.53	4.35	1	31 157	1	3178.5	1
4-Methylheptane	643	**6.2e-22**	↓	74.14	**8.3e-13**	0.33	1	25 892	**0.012**	2928.5	1
Octane	8566	**1.3e-11**	↑	31.68	**4.1e-04**	9.97	0.4	20 355	**1.9e-10**	3996	1
beta-Pinene	3917	0.18		7.77	1	1.47	1	32 400	1	2961	1
3-Carene	4481.5	0.66		7.42	1	14.91	**0.034**	29 858.5	1	3201	1
Acetone	6981	**0.0067**	↑	25.98	**0.0053**	3.25	1	15 789	**2.8e-20**	2120	**0.0015**
2-Butanone	10 165	**1.3e-25**	↑	36.92	**3.7e-05**	11.65	0.17	20 717	**8.5e-10**	2219	**0.0057**
2,3-Butanedione	1745	**1.5e-12**	↓	3.76	1	8.48	0.85	13 647	**6.0e-26**	2807	1
2-Pentanone	10 265	**1.1e-26**	↑	4.48	1	12.45	0.12	29 367	1	5158	**1.2e-05**
3-Pentanone	9798	**6.1e-22**	↑	8.88	1	14.49	**0.042**	18 116	**7.0e-15**	4173	1
2-Hexanone	10 362	**1.0e-27**	↑	12.09	1	13.82	0.059	46 729.5	**9.2e-16**	5715	**2.5e-10**
4-Methyl-2-pentanone	7054	**0.0034**	↑	8.79	1	5.63	1	27 663	0.46	4004.5	1
2-Heptanone	10 320	**2.9e-27**	↑	7.26	1	14.14	0.05	48 373	**1.4e-19**	5641.5	**1.2e-09**
2-Octanone	10 352	**1.3e-27**	↑	10.72	1	13.8	0.059	44 031	**2.4e-10**	5562	**6.5e-09**
2-Methyl-propanenitrile	2081	**3.5e-10**	↓	5.02	1	18.37	**0.0061**	26 075	**0.019**	2770	1
2-Methyl-butanenitrile	1670	**4.1e-13**	↓	5.56	1	1.29	1	26 202.5	**0.025**	3715	1
Dimethyl disulfide	10 241	**2.0e-26**	↑	13.47	1	9.69	0.46	39 850	**4.5e-04**	3533	1

Table S4. Continued from previous page.

Compound	Difference between incubation periods (4-6 and 16-20 weeks) Mann-Whitney U statistic	p-value (adj.)	inc./ dec.	Differences between inoculation days, 4-6 weeks: K.-W. rank sum statistic (5 d.f.)	p-value (adj.)	16-20 weeks: K.-W. rank sum statistic (2 d.f.)	p-value (adj.)	Difference between control vials and actual samples, 4-6 weeks: Mann-Whitney U statistic	p-value (adj.)	16-20 weeks: Mann-Whitney U statistic	p-value (adj.)
Chloroform	1055	**3.9e-18**	↓	6.52	1	2.7	1	27 041.5	0.14	2599	0.39
Bromodichloro-methane	356	**8.7e-25**	↓	6.86	1	0.62	1	32 602.5	1	2989	1
Ethyl tert-butyl ether	255	**5.6e-26**	↓	134.44	**1.6e-25**	22.57	**7.4e-04**	36 044	1	3599	1

Table S5. Statistical comparisons of MAP-negative test tubes with control vials prepared at the same day (adj.: adjusted via Benjamini-Hochberg procedure for 248 tests). Significant differences indicate VOC emissions from sample material. Control vials for cattle feces samples showed unexpectedly low concentration values for 1-propanol (*). In comparison to other control vials incubated for 4, 6 or 8 weeks, cattle feces samples did not show a significant increase in 1-propanol.

	cattle tissue		goat tissue		cattle feces		goat feces	
Compound	Mann-Whitney U statistic	p-value (adj.)	Mann-Whitney U statistic	p-value (adj.)	Mann-Whitney U statistic	p-value (adj.)	Mann-Whitney U statistic	p-value (adj.)
Ethanol	1260	**0.012**	984	0.71	1305	**0.0035**	934	0.8
1-Propanol	1319	**0.0029**	1580	**3.7e-07**	1800	**(5.3e-13)***	876	1
2-Propanol	1135	0.14	1040	0.44	1429	**1.5e-04**	969	0.65
2-Butanol	935	0.95	1160	0.1	1177	0.077	568	1
2-Methyl-1-propanol	821.5	1	902	1	871.5	1	270	1
2-Methyl-1-butanol	900	1	1200	**0.0029**	900	1	870	1
3-Methyl-1-butanol	900	1	1200	**0.0029**	900	1	870	1
1-Pentanol	911	1	830	1	726	1	377	1
1-Hexanol	886.5	1	1099	0.24	830	1	334.5	1
1-Octen-3-ol	868	1	765	1	810	1	583	1
Acetaldehyde	1258	**0.012**	1404	**3.4e-04**	1158	0.1	940	0.8
Propanal	1379	**6.5e-04**	995	0.65	1451	**1.0e-04**	1042	0.31
2-Methylpropanal	1444	**1.0e-04**	1141	0.13	1364	**9.0e-04**	1067	0.23
2-Methylbutanal	1095	0.24	740	1	699	1	642	1
3-Methylbutanal	874	1	619	1	654	1	401	1
Pentanal	852	1	583	1	667	1	337	1
Hexanal	787	1	679	1	631	1	353	1
Benzaldehyde	1117	0.18	532	1	674	1	471	1
Heptanal	735	1	623.5	1	574	1	252	1
Octanal	861	1	527	1	591	1	329	1
Nonanal	949	0.89	544.5	1	682	1	281	1
Furan	1372	**7.5e-04**	1042.5	0.44	1111	0.2	951.5	0.71
2-Methylfuran	897	1	892	1	756	1	778	1
3-Methylfuran	968	0.8	826	1	766	1	870	1
2-Ethylfuran	749	1	554	1	519	1	173	1
2,3,5-Trimethylfuran	892	1	790	1	422	1	282	1
2-Propylfuran	823	1	558	1	563	1	164	1
2-Butylfuran	735	1	616	1	543	1	126	1
2-Pentylfuran	873	1	653	1	609	1	161	1
Isoprene	900	1	990	0.21	900	1	870	1
2-Methylbutane	837	1	1140	0.13	873	1	906	0.95
Pentane	997	0.65	1160	0.1	820	1	1024	0.38
Benzene	861	1	722	1	360	1	827	1
2-Methyl-1-pentene	965	0.8	876	1	629	1	633	1
Cyclohexane	686	1	1173	0.081	1330	**0.0027**	1275.5	**0.0029**
2,2-Dimethylbutane	937	0.95	930	0.97	919	1	965	0.65
2,3-Dimethylbutane	908	1	1044	0.44	970	0.8	991.5	0.52
2-Methylpentane	832	1	970	0.8	999	0.65	1104	0.13
3-Methylpentane	855	1	937	0.95	935	0.95	920	0.89
Hexane	988	0.69	809	1	870	1	734	1
Toluene	645	1	1053	0.4	771	1	728	1
Styrene	825.5	1	1095	0.24	901.5	1	687	1
Ethylbenzene	762	1	1068.5	0.33	912.5	1	840	1
4-Methylheptane	906	1	1319	**0.0029**	836	1	1002.5	0.47
Octane	915	1	807	1	840	1	501	1
beta-Pinene	660	1	1044	0.42	837	1	961.5	0.65
3-Carene	814	1	1015.5	0.44	920.5	0.97	1000.5	0.1
Acetone	1395	**4.1e-04**	1058	0.38	1281	**0.007**	986	0.55
2-Butanone	1177	0.077	896	1	1157	0.1	1145	0.075
2,3-Butanedione	1128	0.15	1315	**0.0029**	1200	0.051	819	1
2-Pentanone	832	1	846	1	837	1	408	1
3-Pentanone	792	1	1076	0.31	877	1	406	1
2-Hexanone	905	1	764	1	709	1	351	1
4-Methyl-2-pentanone	790	1	965	0.8	1029	0.5	714	1
2-Heptanone	891	1	712	1	659	1	383	1
2-Octanone	904	1	819	1	683	1	430	1
2-Methylpropanenitrile	1209.5	**0.042**	1083	0.29	1070	0.33	1178	**0.038**
2-Methylbutanenitrile	952	0.89	1074	0.32	1013	0.58	682	1
Dimethyl disulfide	996	0.65	1132	0.14	1185	0.071	964	0.65
Chloroform	1327	**0.0027**	946	0.91	1088	0.27	1102	0.14
Bromodichloromethane	1066	0.34	951.5	0.89	1025	0.52	985	0.55
Ethyl tert-butyl ether	754	1	93	1	874	1	876.5	1

Table S6. Evaluation metrics for both analyses (targeting MAP presence or MAP growth scores) and all four settings (cattle or goat, feces or tissue). The number of selected variables results from the variable selection step via Boruta. The parameter mtry denotes the number of variables that are randomly drawn as candidates for a split rule for building a random forest classifier. This parameter was optimized in advance on a subset of the available data. The cross-validated accuracy refers to the averaged classification accuracy of ten repeats of ten-fold cross validation.

classification target	setting	number of selected variables	mtry	cross-validated accuracy
MAP presence	cattle feces	30	16	0.984
	cattle tissue	29	2	1.000
	goat feces	24	2	0.889
	goat tissue	18	2	0.901
MAP growth score	cattle feces	36	2	0.886
	cattle tissue	33	2	0.839
	goat feces	32	2	0.838
	goat tissue	26	2	0.819

Table S7. Confusion matrices summarize the distribution of misclassified samples, as below for random forest analyses targeting MAP growth scores for cattle feces samples. The results are averaged across ten times repeated ten-fold cross-validation runs. For example, 8.2 of 11 samples of growth score 0 were on average classified correctly, whereas 1.8 and 1.0 samples were classified as MAP-negative and score 0.5, respectively. Numbers unequal to zero are highlighted in bold.

		Prediction						
		MAP-negative	score 0	score 0.5	score 1	score 2	score 3	score 4
Reference	MAP-negative ($n = 60$)	**60.0**	0.0	0.0	0.0	0.0	0.0	0.0
	MAP growth score 0 ($n = 11$)	**1.8**	**8.2**	**1.0**	0.0	0.0	0.0	0.0
	MAP growth score 0.5 ($n = 10$)	0.0	**1.0**	**8.0**	**1.0**	0.0	0.0	0.0
	MAP growth score 1 ($n = 11$)	0.0	0.0	**1.3**	**8.4**	0.0	**1.3**	0.0
	MAP growth score 2 ($n = 4$)	0.0	0.0	0.0	0.0	**1.7**	**1.4**	**0.9**
	MAP growth score 3 ($n = 8$)	0.0	0.0	0.0	**1.4**	**0.2**	**4.6**	**1.8**
	MAP growth score 4 ($n = 14$)	0.0	0.0	0.0	0.0	0.0	**0.5**	**13.5**

Table S8. Confusion matrix from random forest analyses targeting MAP growth scores for cattle tissue samples, averaged across ten times repeated ten-fold cross-validation runs (for details see Table S7).

		Prediction					
		MAP-negative	score 0.5	score 1	score 2	score 3	score 4
Reference	MAP-negative ($n = 60$)	**60.0**	0.0	0.0	0.0	0.0	0.0
	MAP growth score 0.5 ($n = 11$)	0.0	**5.8**	**4.4**	**0.8**	0.0	0.0
	MAP growth score 1 ($n = 18$)	0.0	**2.9**	**12.7**	**2.1**	**0.3**	0.0
	MAP growth score 2 ($n = 10$)	0.0	0.0	**3.0**	**4.0**	**3.0**	0.0
	MAP growth score 3 ($n = 9$)	0.0	0.0	**0.7**	**1.0**	**7.1**	**0.2**
	MAP growth score 4 ($n = 12$)	0.0	0.0	0.0	0.0	**1.0**	**11.0**

Table S9. Confusion matrix from random forest analyses targeting MAP growth scores for goat feces samples, averaged across ten times repeated ten-fold cross-validation runs (for details see Table S7). Information on the sample with score 0.5 could not be restored due to numerical reasons.

		Prediction				
		MAP-negative	score 0	score 0.5	score 3	score 4
Reference	MAP-negative ($n = 60$)	**55.56**	**5.06**	0.00	**0.11**	0.00
	MAP growth score 0 ($n = 39$)	**9.56**	**29.69**	0.00	**0.11**	0.00
	MAP growth score 0.5 ($n = 1$)	0.00	0.00	0.00	0.00	0.00
	MAP growth score 3 ($n = 12$)	0.00	0.00	0.00	**10.91**	**1.12**
	MAP growth score 4 ($n = 8$)	0.00	0.00	0.00	**3.49**	**4.39**

Table S10. Confusion matrix from random forest analyses targeting MAP growth scores for goat tissue samples, averaged across ten times repeated ten-fold cross-validation runs (for details see Table S7).

		Prediction						
		MAP-negative	score 0	score 0.5	score 1	score 2	score 3	score 4
Reference	MAP-negative ($n = 60$)	**58.8**	**0.4**	**0.8**	0.0	0.0	0.0	0.0
	MAP growth score 0 ($n = 11$)	**8.3**	**1.7**	**1.0**	0.0	0.0	0.0	0.0
	MAP growth score 0.5 ($n = 11$)	**1.6**	**0.3**	**8.7**	**0.4**	0.0	0.0	0.0
	MAP growth score 1 ($n = 3$)	0.0	0.0	**1.0**	0.0	**2.0**	0.0	0.0
	MAP growth score 2 ($n = 4$)	0.0	0.0	0.0	**1.0**	**1.2**	**1.8**	0.0
	MAP growth score 3 ($n = 25$)	0.0	0.0	0.0	0.0	0.0	**23.8**	**1.2**
	MAP growth score 4 ($n = 6$)	0.0	0.0	0.0	0.0	0.0	**2.2**	**3.8**
